# Adiposity assessed close to diagnosis and prostate cancer prognosis in the EPIC study

**DOI:** 10.1093/jncics/pkae070

**Published:** 2024-08-24

**Authors:** Margarita Cariolou, Sofia Christakoudi, Marc J Gunter, Tim Key, Aurora Pérez-Cornago, Ruth Travis, Raul Zamora-Ros, Kristina Elin T Petersen, Anne Tjønneland, Elisabete Weiderpass, Rudolf Kaaks, Petra Seibold, Elif Inan-Eroglu, Matthias B Schulze, Giovanna Masala, Claudia Agnoli, Rosario Tumino, Chiara Di Girolamo, Amaia Aizpurua, Miguel Rodriguez-Barranco, Carmen Santiuste, Marcela Guevara, Dagfinn Aune, Doris S M Chan, David C Muller, Konstantinos K Tsilidis

**Affiliations:** Department of Epidemiology and Biostatistics, School of Public Health, Faculty of Medicine, Imperial College London, London, UK; Department of Epidemiology and Biostatistics, School of Public Health, Faculty of Medicine, Imperial College London, London, UK; Department of Epidemiology and Biostatistics, School of Public Health, Faculty of Medicine, Imperial College London, London, UK; Cancer Epidemiology Unit, Nuffield Department of Population Health, University of Oxford, Oxford, UK; Cancer Epidemiology Unit, Nuffield Department of Population Health, University of Oxford, Oxford, UK; Cancer Epidemiology Unit, Nuffield Department of Population Health, University of Oxford, Oxford, UK; Unit of Nutrition and Cancer, Cancer Epidemiology and Research Programme, Catalan Institute of Oncology (ICO), Bellvitge Biomedical Research Institute (IDIBELL), Barcelona, Spain; Danish Cancer Institute, Diet, Cancer and Health, Copenhagen, Denmark; Danish Cancer Institute, Diet, Cancer and Health, Copenhagen, Denmark; Department of Public Health, University of Copenhagen, Copenhagen , Denmark; International Agency for Research on Cancer, Lyon, France; Division of Cancer Epidemiology, German Cancer Research Center, Heidelberg, Germany; Division of Cancer Epidemiology, German Cancer Research Center, Heidelberg, Germany; Department of Molecular Epidemiology, German Institute of Human Nutrition Potsdam-Rehbruecke, Nuthetal, Germany; Department of Molecular Epidemiology, German Institute of Human Nutrition Potsdam-Rehbruecke, Nuthetal, Germany; Institute of Nutritional Science, University of Potsdam, Nuthetal, Germany; Clinical Epidemiology Unit, Institute for Cancer Research, Prevention, and Clinical Network, Florence, Italy; Epidemiology and Prevention Unit, IRCCS National Cancer Institute Foundation, Milan, Italy; Hyblean Association for Epidemiological Research, AIRE ONLUS Ragusa, Ragusa, Italy; Centre for Biostatistics, Epidemiology, and Public Health, Department of Clinical and Biological Sciences, University of Turin, Orbassano, Italy; Ministry of Health of the Basque Government, Sub directorate for Public Health and Addictions of Gipuzkoa, San Sebastián, Spain; Biodonostia Health Research Institute, Epidemiology of Chronic and Communicable Diseases Group, San Sebastián, Spain; Escuela Andaluza de Salud Pública, Granada, Spain; Instituto de Investigación Biosanitaria ibs. GRANADA, Granada, Spain; Centro de Investigación Biomédica en Red de Epidemiología y Salud Pública, Madrid, Spain; Centro de Investigación Biomédica en Red de Epidemiología y Salud Pública, Madrid, Spain; Department of Epidemiology, Murcia Regional Health Council, Murcia-IMIB, Spain; Centro de Investigación Biomédica en Red de Epidemiología y Salud Pública, Madrid, Spain; Instituto de Salud Pública y Laboral de Navarra, Pamplona, Spain; Navarra Institute for Health Research, Pamplona, Spain; Department of Epidemiology and Biostatistics, School of Public Health, Faculty of Medicine, Imperial College London, London, UK; Department of Nutrition, Oslo New University College, Oslo, Norway; Department of Research, Cancer Registry of Norway, Norwegian Institute of Public Health, Oslo, Norway; Department of Epidemiology and Biostatistics, School of Public Health, Faculty of Medicine, Imperial College London, London, UK; Department of Epidemiology and Biostatistics, School of Public Health, Faculty of Medicine, Imperial College London, London, UK; Department of Epidemiology and Biostatistics, School of Public Health, Faculty of Medicine, Imperial College London, London, UK; Department of Hygiene and Epidemiology, University of Ioannina Medical School, Ioannina, Greece

## Abstract

**Background:**

Adiposity has been characterized as a modifiable risk factor for prostate cancer. Its association with outcomes after prostate cancer diagnosis, however, must be better understood, and more evidence is needed to facilitate the development of lifestyle guidance for patients with prostate cancer.

**Methods:**

We investigated the associations between adiposity indices close to prostate cancer diagnosis (up to 2 years before or up to 5 years after diagnosis) and mortality in 1968 men of the European Prospective Investigation into Cancer and Nutrition cohort. Men were followed up for a median of 9.5 years. Cox proportional hazards models were adjusted for age and year of diagnosis, disease stage and grade, and smoking history and stratified by country.

**Results:**

Each 5-unit increment in prediagnosis or postdiagnosis body mass index combined was associated with a 30% higher rate of all-cause mortality and a 49% higher rate of prostate cancer–specific mortality. Similarly, each 5-unit increment in prediagnosis body mass index was associated with a 35% higher rate of all-cause mortality and a 51% higher rate of prostate cancer–specific mortality. The associations were less strong for postdiagnosis body mass index, with a lower number of men in analyses. Less clear positive associations were shown for waist circumference, hip circumference, and waist to hip ratio, but data were limited.

**Conclusions:**

Elevated levels of adiposity close to prostate cancer diagnosis could lead to higher risk of mortality; therefore, men are encouraged to maintain a healthy weight. Additional research is needed to confirm whether excessive adiposity after prostate cancer diagnosis could worsen prognosis.

Prostate cancer is a major public health burden challenging the economic and health-care systems of many countries globally (1,2). It is the second-most frequently diagnosed malignancy after lung cancer and the fifth-leading cause of cancer-related deaths in men worldwide (1.7 million cases and 500 000 annual deaths expected by 2030) (3-5). Most men have a diagnosis of localized prostate cancer and up to 99% 10-year survival (6), whereas the 5-year survival for individuals with late-stage disease is approximately 30% (6). Survival rates have substantially improved (5,7,8), but extended survival often coexists with increased cancer-related comorbidities, including obesity (9).

General adiposity (body mass index [BMI]) has been inversely associated with the risk of developing localized and total prostate cancer (10-12); general adiposity (BMI) and abdominal adiposity (waist circumference, waist to hip ratio) have been positively associated with the risk of developing aggressive (10,11,13-16) and fatal prostate cancer (10,17) in studies, including European Prospective Investigation into Cancer and Nutrition (EPIC). The literature on potential associations between modifiable factors and prostate cancer prognosis is inconclusive (18-20). Currently, any existing lifestyle survivorship guidance is mainly extrapolated from cancer prevention recommendations. Individuals with cancer are advised to avoid obesity and adhere to a balanced lifestyle (20,21). A recent meta-analysis of observational studies reported evidence of a J-shaped association between BMI assessed at or after prostate cancer diagnosis and all-cause mortality (17 studies) and a similar association for prostate cancer–specific mortality (13 studies). No associations were identified between waist circumference and mortality (3 studies) (19). Observational studies that explored adiposity close to diagnosis in relation to prostate cancer survival are subject to limitations, such as being single centered, using a single-timepoint adiposity measure instead of measures at different timepoints throughout the cancer survivorship trajectory, focused on general adiposity without incorporating measures of abdominal or gluteofemoral (hip circumference) adiposity. A few studies investigated potential nonlinear relationships (22), but none explored stage-specific or grade-specific associations (19). Investigating the association between obesity and cancer-related survival outcomes should be among the top research priorities (23). We explored the associations among general, abdominal, and gluteofemoral adiposity assessed close to diagnosis with mortality in the EPIC study.

## Methods

**Figure 1.**
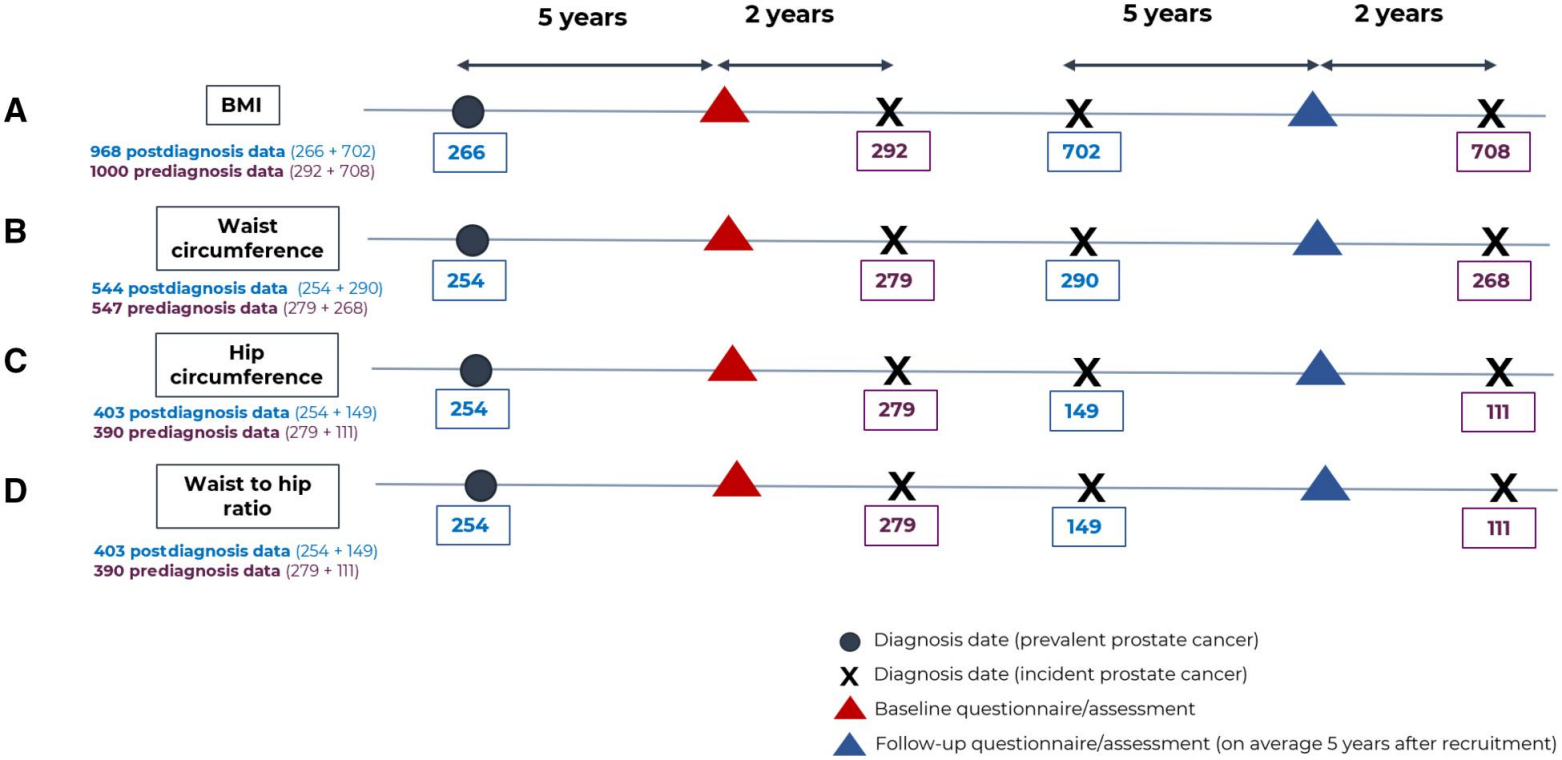
Data collection time frames showing the total number of men who had data on adiposity close to diagnosis for (**A**) BMI, (**B**) waist circumference, (**C**) hip circumference, and (**D**) waist to hip ratio. The prediagnosis analyses included men with incident prostate cancer diagnosed during cohort follow-up who had baseline (at recruitment) or follow-up anthropometric data collected up to 2 years before diagnosis. The postdiagnosis analyses included men with prevalent prostate cancer at study entry who had baseline anthropometric data collected up to 5 years after diagnosis or men with incident prostate cancer who had follow-up anthropometric data collected up to 5 years after diagnosis. The numbers shown on the diagram represent the total number of men with prostate cancer before exclusion of individuals with missing data on the covariates of the main model of the present study (ie, age at diagnosis, year of diagnosis, disease stage, tumor grade, and smoking status). BMI = body mass index.

EPIC is a multicenter prospective cohort study that recruited volunteers aged 35-70 years between 1992 and 2000. The final cohort included 153 457 men. Study design and data-collection procedures are detailed elsewhere (24). Eligibility criteria, identification of prostate cancer cases, and outcome assessment are described in detail in the Supplementary Methods (available online). Briefly, men with prostate cancer were eligible for inclusion if they had adiposity data collected close to diagnosis (ie, up to 2 years before [prediagnosis] or up to 5 years after [postdiagnosis]) (Figure 1, A-D). Data were used from all EPIC centers apart from France, Norway, Utrecht, and Naples, which recruited only women. Data from Greece were not available for this analysis. The *International Classification of Diseases for Oncology, 3rd Edition* (code C61.9) was used to select individuals with primary malignant prostate tumors (25).

Tumor grade and stage information at diagnosis was collected, if possible, from each center. Height, weight, waist, and hip circumference were obtained in all centers apart from Umea, which had information only about height and weight. Height, weight, hip, and waist circumference were measured using standard protocols in all participating EPIC centers in this study; only in Oxford, United Kingdom, were they self-reported. A follow-up assessment on average 5 years after recruitment collected data from 350 000 participants (28% men). Weight was self-reported in all centers apart from Norfolk, United Kingdom, where it was measured (24,26). At study entry, participants provided information about lifestyle, previous diseases, alcohol and tobacco consumption, exercise, diet, and sociodemographics. Supplementary Methods (available online) provides details of how important variables were defined.

### Statistical analysis

Cox proportional hazards regression models were used to estimate hazard ratios (HRs) and 95% confidence intervals (CIs) for the associations between adiposity assessed either up to 2 years before diagnosis or up to 5 years after diagnosis in relation to all-cause and prostate cancer–specific mortality. Analyses were also performed by combining both time frames. The date of the at-recruitment or follow-up anthropometric assessment/questionnaire was considered the start of follow-up, and the date of death, emigration, withdrawal or loss to follow-up or last follow-up, whichever occurred first, was the end of follow-up. In the analysis of prostate cancer–specific mortality; all other deaths were censored. We obtained estimates of the linear associations between adiposity variables and mortality per 5 kg/m^2^ increments in BMI, 10-cm increments in waist and hip circumference, and 0.1-unit increments in waist to hip-ratio. Restricted cubic splines were also used to investigate possible nonlinear associations, with 3 knots at the 10th, 50th, and 90th percentiles of the adiposity variable distribution (27). Nonlinearity was evaluated graphically and using the likelihood ratio test, comparing models with and without the restricted cubic spline term (28-30). The median value of each adiposity variable in each analysis was used as the referent.

Nested multivariable models were adjusted for relevant covariates selected a priori, on the basis of subject matter knowledge, including age and year of diagnosis, Gleason score/grade, tumor stage, prostate-specific antigen level, smoking status, and physical activity (Supplementary Table 1, available online). Model 1 was adjusted for age and year of diagnosis. Model 2 was additionally adjusted for disease stage and grade. Model 3, which represented our main model and is the one presented in our results, was additionally adjusted for smoking status. A fourth model, additionally adjusted for lifetime number of cigarettes per day, physical activity, and prostate-specific antigen level, resulted in similar results but a considerably larger percentage of missing data. All models were stratified by country to account for potential center-specific differences, including follow-up procedures and questionnaires (26,31). We performed complete-case analyses restricted to individuals with complete information about the covariates of each model (32,33). The number of men and deaths per model are shown in Supplementary Table 2 (available online). To examine the influence of covariates, we performed sensitivity analyses, including in models 1 and 2 the same participants as in model 3 (the main model). As a proxy for socioeconomic status, the prediagnosis or postdiagnosis BMI combined model was additionally adjusted for highest school level. The postdiagnosis models were also adjusted for prediagnosis BMI in sensitivity analysis. Analyses were conducted by the World Health Organization (WHO) BMI categories, but inference was the same (Supplementary Tables 3 and 4, available online).

Graphical inspection of the smoothed scaled Schoenfeld residuals showed no violation of the proportional hazards assumption (34-36). Sensitivity analyses were performed to assess potential reverse causation and selection bias (details in Supplementary Methods, available online). We also performed analyses by tumor stage and grade at diagnosis, smoking status, and year of BMI measurement (1 and 2 years before diagnosis and for each year after diagnosis) because each year may reflect differences in the cancer care continuum. A *P* value of .05 was considered statistically significant; statistical tests were 2 sided. R, version 4.0.5, software (R Foundation for Statistical Computing, Vienna, Austria) was used for analyses.

## Results

### Cohort characteristics

A total of 1968 men with prostate cancer had adiposity data collected up to 2 years before or up to 5 years after diagnosis. Important tumor and lifestyle characteristics of these men are shown in Table 1. Some data were missing for prostate-specific antigen (45%), tumor stage (39%), tumor grade (34%), lifetime cigarettes per day (31%), physical activity (15%), and smoking status (4%), but all men had complete information for age and year of diagnosis (Supplementary Table 5, available online). Tumor and baseline lifestyle characteristics of the men with prostate cancer who were not included in this study (if they did not have adiposity data close to diagnosis) were not materially different from those of the men who were included. Important characteristics of those included in the main BMI analyses (after removing men with missing covariate data) were comparable to the characteristics of the total number of men who had BMI data close to diagnosis (with missing data) (Supplementary Table 6, available online). Key characteristics of the 1968 men were also largely similar by the World Health Organization categories (Supplementary Table 7, available online).

**Table 1.** Tumor and lifestyle characteristics of the men diagnosed with prostate cancer who had prediagnosis or postdiagnosis adiposity data^a^

	BMI (N = 1968)	Waist circumference (n = 1091)	Hip circumference (n = 793)	Waist to hip ratio (n = 793)
Follow-up time (ie, from return of either baseline or follow-up questionnaire until censoring), median (5th-95th percentile), y	9.5 (2.0-17.6)	8.4 (2.0-18.3)	7.6 (2.0-18.8)	7.6 (2.0-18.8)
Age at diagnosis, median (5th-95th percentile), y	66 (55-77)	65 (54-74)	65 (54-75)	65 (54-75)
Disease stage—EPIC stage classification, No. (%)				
In situ	0 (0)	0 (0)	0 (0)	0 (0)
Localized	710 (36)	308 (28)	116 (15)	116 (15)
Metastatic	49 (3)	6 (1)	6 (1)	6 (1)
Metastatic, regional	77 (4)	41 (4)	18 (2)	18 (2)
Metastatic, distant	56 (3)	52 (5)	17 (2)	17 (2)
Unknown/missing	1076 (55)	684 (63)	636 (80)	636 (80)
Tumor stage—TNM code or, if TNM not available, EPIC stage classification , No. (%)				
Localized (T0-T2 and N0-NX and M0)	824 (42)	314 (29)	123 (16)	123 (16)
Advanced (T3-T4 and/or N1-N3 or M1)	382 (19)	191 (18)	129 (16)	129 (16)
Unknown/missing	762 (39)	586 (54)	541 (68)	541 (68)
Tumor grade—EPIC grade classification, No. (%)				
Well differentiated	58 (3)	24 (2)	24 (3)	24 (3)
Moderately differentiated	282 (14)	119 (11)	119 (15)	119 (15)
Poor/undifferentiated	91 (5)	280 (26)	40 (5)	40 (5)
Unknown/missing	1537 (78)	668 (61)	610 (77)	610 (77)
Tumor grade—Gleason score or, if not available, EPIC grade classification, No. (%)				
Gleason score 2-6 (well differentiated)	632 (32)	272 (25)	156 (20)	156 (20)
Gleason score 7 (moderately differentiated)	456 (23)	208 (19)	146 (18)	146 (18)
Gleason score 8-10 (poorly or undifferentiated)	211 (11)	117 (11)	65 (8)	65 (8)
Unknown/undetermined	669 (34)	494 (45)	426 (54)	426 (54)
Tumor grade—Gleason score, otherwise EPIC grade classification (Gleason score 7 [moderately differentiated] as a separate category and split into 3 + 4 or 4 + 3), No. (%)				
Gleason score 2-6 (well differentiated)	632 (32)	272 (25)	156 (20)	156 (20)
Gleason score 7 (3 + 4) (moderately differentiated)	199 (10)	91 (8)	50 (6)	50 (6)
Gleason score 7 (4 + 3) (moderately differentiated)	78 (4)	22 (2)	18 (2)	18 (2)
Gleason score 8-10 (poorly or undifferentiated)	211 (11)	117 (11)	65 (8)	65 (8)
Unknown/undetermined	848 (43)	589 (54)	504 (64)	504 (64)
**Anthropometry**				
BMI				
BMI, median (5th-95th percentile)	26 (22-33)	26 (22-33)	26 (22-33)	26 (22-33)
Underweight (<18.5), No. (%)	8 (0)	5 (0)	5 (1)	5 (1)
Normal weight (18.5-24.9), No. (%)	643 (33)	363 (33)	262 (33)	262 (33)
Overweight (25-29.9), No. (%)	1037 (52)	572 (52)	414 (52)	414 (52)
Obese (≥30), No. (%)	280 (14)	151 (14)	112 (14)	112 (14)
Unknown, No. (%)	—^b^	—^b^	—^b^	—^b^
Waist circumference				
Waist circumference, median (5th-95th percentile)	97 (82-115)	97 (82-115)	97 (81-115)	97 (81-115)
Unknown, No. (%)	877 (45)	—^b^	—^b^	—^b^
Hip circumference				
Hip circumference, median (5th-95th percentile)	101 (90-114)	101 (90-114)	101 (90-114)	101 (90-114)
Unknown, No. (%)	1175 (60)	298 (27)	—^b^	—^b^
Waist to hip ratio				
Waist to hip ratio, median (5th-95th percentile)	1 (0.85-1.06)	0.96 (0.85-1.06)	0.96 (0.85-1.06)	0.96 (0.85-1.06)
Unknown, No. (%)	1175 (60)	298 (27)	—^b^	—^b^
Smoking status, No. (%)				
Never smoker	645 (33)	334 (31)	243 (31)	243 (31)
Former smoker	979 (50)	537 (49)	397 (50)	397 (50)
Current smoker	270 (14)	182 (17)	118 (15)	118 (15)
Unknown	74 (4)	38 (3)	35 (4)	35 (4)
Cambridge Physical Activity Index, No. (%)				
Inactive	495 (25)	284 (26)	215 (27)	215 (27)
Moderately inactive	591 (30)	396 (36)	298 (38)	298 (38)
Moderately active	322 (16)	197 (18)	126 (16)	126 (16)
Active	256 (13)	184 (17)	124 (16)	124 (16)
Missing	304 (15)	30 (3)	30 (4)	30 (4)
Highest school level (baseline), No. (%)				
None	29 (2)	6 (1)	6 (1)	6 (1)
Primary school completed	657 (33)	364 (33)	266 (34)	266 (34)
Technical/professional school	433 (22)	249 (23)	174 (22)	174 (22)
Secondary school	224 (11)	140 (13)	116 (15)	116 (15)
Longer education (including university degree)	525 (27)	285 (26)	185 (23)	185 (23)
Not specified	78 (4)	35 (3)	35 (4)	35 (4)
Unknown	22 (1)	12 (1)	11 (1)	11 (1)
Prostate-specific antigen level, median (5th-95th percentile)	11 (3-134)	12 (3-249)	11 (2-166)	11.1 (2-166)

a Percentages are rounded to the nearest whole number. The characteristics of individuals are taken from each respective time frame (either baseline or follow-up) for which the individual was selected according to the eligibility criteria of this study (ie, for any variables that also had follow-up measurements). BMI = body mass index; EPIC = European Prospective Investigation into Cancer and Nutrition.

b Unknown or missing data.

### Main results: BMI and mortality

**Figure 2.**
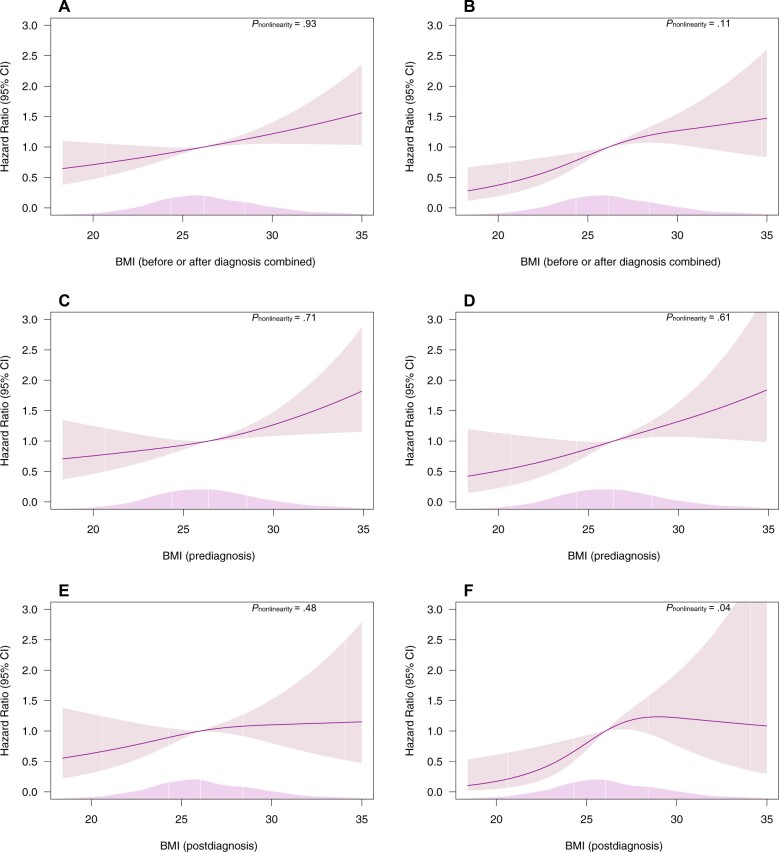
Hazard ratios from Cox proportional hazards models with restricted cubic spline curves describing the association between BMI data collected before or after diagnosis combined and (**A**) all-cause mortality (men/deaths = 972/320); (**B**) prostate cancer–specific mortality (men/deaths = 972/163), prediagnosis BMI; (**C**) all-cause mortality (men/deaths = 570/194); (**D**) prostate cancer–specific mortality (men/deaths = 570/100), postdiagnosis BMI; (**E**) all-cause mortality (men/deaths = 372/126); (**F**) prostate cancer–specific mortality (men/deaths = 372/63). Hazard ratios are based on the main model, adjusted for age at diagnosis, year of diagnosis, disease stage, tumor grade, and smoking status; knots at the 10th, 50th, and 90th percentiles of BMI. The median BMI of the individuals included in analyses was used as the referent: 26.2 in the prediagnosis or postdiagnosis BMI analysis, 26.5 in the prediagnosis BMI analysis, and 25.9 in the postdiagnosis BMI analysis. The smooth density plot represents the density of the population across the spline variable. BMI = body mass index; CI = confidence interval.

The 1968 men with BMI data collected either up to 2 years before diagnosis (n = 1000) or up to 5 years after diagnosis (n = 968) (Figure 1, A; Supplementary Figure 1, A, available online) were followed for a median of 9.5 years from return of the baseline or follow-up questionnaire. Analysis of BMI assessed before or after diagnosis combined showed a linear increase in the rate of all-cause (HR per 5 kg/m^2^ = 1.30, 95% CI = 1.11 to 1.52, *P*_nonlinearity_ = .93) and prostate cancer–specific mortality (HR per 5 kg/m^2^ = 1.49, 95% CI = 1.21 to 1.84, *P*_nonlinearity_ = .11) (Table 2; Figure 2, A and B). A positive association was also observed between prediagnosis BMI and all-cause mortality (HR per 5 kg/m^2^ = 1.35, 95% CI = 1.11 to 1.65, *P*_nonlinearity_ = .71) as well as prostate cancer–specific mortality (HR per 5 kg/m^2^ = 1.51, 95% CI = 1.16 to 1.96, *P*_nonlinearity_ = .61) (Table 2; Figure 2, C and D). For postdiagnosis BMI and all-cause mortality, the 95% confidence intervals were wide, crossing the null (HR per 5 kg/m^2^ = 1.23, 95% CI = 0.91 to 1.66, *P*_nonlinearity_ = .48). Little evidence of nonlinearity was observed for prostate cancer–specific mortality because it was based on few events (*P*_nonlinearity_ = .04) (Table 2; Figure 2, E and F).

### Subgroup and sensitivity analyses for BMI and mortality

Of 1968 men, 42% (n = 824) had localized disease, 19% (n = 382) had advanced disease, 9% (n = 180) had metastatic disease, 39% (n = 762) had missing data, 32% (n = 632) had well-differentiated disease (Gleason scores 2-6), 23% (n = 456) had moderately differentiated disease (Gleason score 7), 11% (n = 211) had poorly or undifferentiated disease (Gleason scores 8-10), and 34% (n = 669) had missing data (Table 1). Positive and similar associations were observed between 5 kg/m^2^ increments of BMI and all-cause and prostate cancer–specific mortality for localized, advanced, and metastatic prostate cancer, after excluding metastatic tumors (Table 2; Supplementary Figure 2, available online), and across tumor grades (Table 2; Supplementary Figure 3, available online). Similar associations to the main analyses were observed upon exclusion of men who had died during the first year of follow-up (Table 2; Supplementary Figure 4, available online). In addition, adjusting the postdiagnosis model for baseline or prediagnosis BMI did not materially change the results (Supplementary Figure 5, available online).

**Table 2.** Cox proportional hazard ratios and 95% confidence intervals for the linear association between adiposity and all-cause and prostate cancer–specific mortality (complete case analyses—main model)

	No. of events/total No. of men with prostate cancer	**Hazard ratio** ^a^ **(95% CI)**	***P*_nonlinearity_** ^b^
**Main analyses**			
BMI (per 5 kg/m^2^)			
All-cause mortality			
Prediagnosis or postdiagnosis combined	320/942	1.30 (1.11 to 1.52)	.93
Prediagnosis	194/570	1.35 (1.11 to 1.65)	.71
Postdiagnosis	126/372	1.23 (0.91 to 1.66)	.48
Prostate cancer–specific mortality			
Prediagnosis or postdiagnosis combined	163/942	1.49 (1.21 to 1.84)	.11
Prediagnosis	100/570	1.51 (1.16 to 1.96)	.61
Postdiagnosis	63/372	1.74 (1.13 to 2.68)	.04
Waist circumference (per 10 cm)			
All-cause mortality			
Prediagnosis or postdiagnosis combined	120/362	1.16 (0.97 to 1.39)	.03
Prediagnosis	86/245	1.31 (1.07 to 1.61)	.06
Postdiagnosis	34/117	0.67 (0.40 to 1.08)	.72
Prostate cancer–specific mortality			
Prediagnosis or postdiagnosis combined	79/362	1.26 (1.01 to 1.57)	.03
Prediagnosis	53/245	1.47 (1.14 to 1.89)	.01
Postdiagnosis	26/117	0.79 (0.46 to 1.37)	.79
Hip circumference (per 10 cm)			
All-cause mortality			
Prediagnosis or postdiagnosis combined	45/167	1.12 (0.72 to 1.77)	.88
Prediagnosis	42/128	1.19 (0.74 to 1.91)	.74
Postdiagnosis	3/39	(Limited data)	—^c^
Prostate cancer–specific mortality			
Prediagnosis or postdiagnosis combined	23/167	1.48 (0.80 to 2.74)	.29
Prediagnosis	21/128	1.88 (0.94 to 3.77)	.62
Postdiagnosis	2/39	(Limited data)	—^c^
Waist to hip ratio (per 0.1 unit)			
All-cause mortality			
Prediagnosis or postdiagnosis combined	45/167	1.18 (0.70 to 1.99)	.47
Prediagnosis	42/128	1.24 (0.71 to 2.15)	.91
Postdiagnosis	3/39	(Limited data)	—^c^
Prostate cancer–specific mortality			
Prediagnosis or postdiagnosis combined	23/167	1.47 (0.67 to 1.02)	.50
Prediagnosis	21/128	1.71 (0.67 to 4.34)	.75
Postdiagnosis	2/39	(Limited data)	—^c^
**Subgroup and sensitivity analyses**			
Prediagnosis or postdiagnosis BMI combined, per 5 kg/m^2^			
Disease stage^d^			
All-cause mortality			
Localized	175/656	1.17 (0.92 to 1.49)	.19
Advanced (includes metastatic)	145/286	1.38 (1.09 to 1.73)	.37
All, excluding metastatic	233/819	1.25 (1.02 to 1.52)	.26
Only metastatic	87/123	1.37 (1.00 to 1.86)	.19
Prostate cancer–specific mortality			
Localized	61/656	1.45 (1.01 to 2.08)	.13
Advanced (includes metastatic)	102/286	1.52 (1.15 to 2.02)	.39
All, excluding metastatic	85/819	1.57 (1.16 to 2.13)	.23
Only metastatic	78/123	1.35 (0.97 to 1.87)	.43
Tumor grade^e^			
All-cause mortality			
Well differentiated—Gleason score 2-6	106/427	1.34 (1.02 to 1.72)	.85
Moderately differentiated—Gleason score 7	118/359	1.20 (0.89 to 1.62)	.12
Poorly differentiated—Gleason score 8-10	96/156	1.21 (0.88 to 1.65)	.22
Prostate cancer–specific mortality			
Well differentiated—Gleason score 2-6	34/427	1.49 (0.99 to 2.27)	.12
Moderately differentiated—Gleason score 7	59/359	1.33 (0.89 to 1.99)	.71
Poorly differentiated—Gleason score 8-10	70/156	1.51 (1.06 to 2.16)	.19
**Additional adjustment for baseline highest school level (proxy for socioeconomic status)**			
All-cause mortality	318/936	1.25 (1.06 to 1.47)	.65
Prostate cancer–specific mortality	163/936	1.43 (1.15 to 1.77)	.07
**Lagged analysis—removing deaths during the first year of follow-up**			
All-cause mortality	308/930	1.34 (1.14 to 1.58)	.90
Prostate cancer–specific mortality	155/930	1.54 (1.25 to 1.91)	.10
**Postdiagnosis BMI analyses, per 5 kg/m^2^**			
Lagged analysis—removing deaths during the first year of follow-up			
All-cause mortality	117/363	1.34 (0.98 to 1.83)	.44
Prostate cancer–specific mortality	57/363	1.96 (1.24 to 3.08)	.04
**Additional adjustment for prediagnosis/baseline BMI**			
All-cause mortality	126/372	1.04 (0.55 to 1.99)	.46
Prostate cancer–specific mortality	63/372	1.49 (0.61 to 3.67)	.03
**By each year of BMI measurement (up to 2 y before diagnosis and up to 5 y after diagnosis), per 5 kg/m^2^**			
First year before diagnosis			
All-cause mortality	78/239	1.07 (0.76 to 1.50)	.52
Prostate cancer–specific mortality	37/239	1.51 (0.91 to 2.50)	.79
Second year before diagnosis			
All-cause mortality	116/331	1.59 (1.24 to 2.04)	.80
Prostate cancer–specific mortality	63/331	1.54 (1.11 to 2.15)	.71
First year after diagnosis			
All-cause mortality	52/146	1.44 (0.84 to 2.46)	.68
Prostate cancer–specific mortality	30/146	1.84 (0.90 to 3.74)	.03
Second year after diagnosis			
All-cause mortality	40/123	1.58 (0.90 to 2.76)	.42
Prostate cancer–specific mortality	20/123	2.09 (0.95 to 4.58)	.93
Third year after diagnosis			
All-cause mortality	26/77	1.05 (0.52 to 2.13)	.67
Prostate cancer–specific mortality	10/77	1.91 (0.49 to 7.47)	.91

a Main model (model 3) hazard ratio adjusted for age, year of diagnosis, disease stage, tumor grade, and smoking status and stratified by EPIC country. BMI = body mass index; CI = confidence interval; EPIC = European Prospective Investigation into Cancer and Nutrition.

b Analysis of variance test of models with the restricted cubic spline term compared with models with the linear term (without the spline term).

c No available data.

d Not adjusted for disease stage apart from the analysis that excludes men with metastatic prostate cancer.

e Not adjusted for tumor grade.

Supplementary Figure 6 (available online) shows the number of men with BMI data at 1 and 2 years before diagnosis and at each year after diagnosis up to 5 years later. During the first year before diagnosis, the association between BMI and all-cause mortality appeared linear (HR per 5 kg/m^2^ = 1.07, 95% CI = 0.76. to 1.50, *P*_nonlinearity_ = .52) and similar for prostate cancer–specific mortality, but the 95% confidence intervals crossed the null (HR per 5 kg/m^2^ = 1.51, 95% CI = 0.91 to 2.50, *P*_nonlinearity_ = .79) (Table 2; Supplementary Figure 7, A and B, available online). A stronger positive association was seen for BMI assessed in the second year before diagnosis with all-cause mortality (HR per 5 kg/m^2^ = 1.59, 95% CI = 1.24 to 2.04, *P*_nonlinearity_ = .80) and prostate cancer–specific mortality (HR per 5 kg/m^2^ = 1.54, 95% CI = 1.11 to 2.15, *P*_nonlinearity_ = .71) (Table 2; Supplementary Figure 7, C and D, available online). There were indications for linearity for the first 3 years after diagnosis (Supplementary Figure 8, available online), but the 95% confidence intervals were wide, crossing the null. Data were scarce beyond the third year after diagnosis (plots not shown).

Stratified analysis by smoking status showed positive associations among never smokers (HR per 5 kg/m^2^ = 1.62, 95% CI = 1.24 to 2.14) and current smokers (HR per 5 kg/m^2^ = 1.45, 95% CI = 1.07 to 1.98) and no association among former smokers (HR per 5 kg/m^2^ = 0.98, 95% CI = 0.76 to 1.28) for all-cause mortality (Supplementary Table 8, Supplementary Figure 9, available online). Additional adjustment for education gave similar positive associations as the main analysis (Table 2; Supplementary Figure 10, available online). Results were similar with individuals in model 3, included in models 1 and 2 (Supplementary Table 9, available online).

### Waist circumference, hip circumference, waist to hip ratio, and mortality

Waist circumference data collected up to 2 years before diagnosis (n = 547) or up to 5 years after diagnosis (n = 544) were available for 1091 of the 1968 men (Figure 1, B; Supplementary Figure 1, B, available online) followed for a median of 8.4 years. Analysis of prediagnosis or postdiagnosis waist circumference combined showed a linear increase in the rate of all-cause mortality (*P*_nonlinearity_ = .03) and prostate cancer–specific mortality (*P*_nonlinearity_ = .03) up to approximately 100 cm, with limited data after this point. Results were similar for prediagnosis waist circumference but less clear in the postdiagnosis analysis because of limited data (Table 2; Supplementary Figure 11, available online). Of 1968 men, 793 had hip circumference and waist to hip ratio data up to 2 years before diagnosis (n = 390) or up to 5 years after diagnosis (n = 403) (Figure 1, C and D; Supplementary Figure 1, C and D, available online), followed for a median of 7.6 years. There were indications of positive associations in the prediagnosis and postdiagnosis groups combined and the prediagnosis analyses for all-cause and prostate cancer–specific mortality, but the 95% confidence intervals were wide, crossing the null (Table 2; Supplementary Figures 12 and 13, available online). Data were scarce and not shown for postdiagnosis hip circumference and waist to hip ratio.

## Discussion

In this study, each 5-unit increment in prediagnosis or postdiagnosis BMI combined was associated with a 30% higher rate of all-cause mortality and a 49% higher rate of prostate cancer–specific mortality, independent of tumor grade, disease stage, smoking status, and other confounders. Each 5-unit increment in prediagnosis BMI was associated with a 35% higher rate of all-cause mortality and a 51% higher rate of prostate cancer–specific mortality. The associations were less strong for postdiagnosis BMI because of a lower number of men in analyses. Data on waist circumference, hip circumference, and waist to hip ratio were more limited than for BMI, but there were indications for positive associations with mortality.

Observational studies in men with prostate cancer that investigated the associations between postdiagnosis adiposity and mortality found inconsistent results. Some (18,22,37) reported positive associations and others reported inverse associations (38-41) between BMI and mortality outcomes. Most linear dose-response meta-analyses in patients with prostate cancer reported no associations (95% CIs crossing the null) between at diagnosis/postdiagnosis BMI (42-44) and prostate cancer–specific or all-cause mortality; only 1 study reported a small increase in the rate of all-cause mortality (44). Our meta-analysis of 2023 identified a J-shaped association between postdiagnosis BMI and all-cause and prostate cancer–specific mortality. Most studies adjusted for stage, tumor grade, and treatment but not for smoking status (19). In this study, we observed less clear associations for BMI assessed up to 5 years after diagnosis with mortality (only 372 men in this analysis). Little evidence of nonlinearity was seen for prostate cancer–specific mortality on the basis of few events, but we observed positive associations between BMI assessed up to 2 years before diagnosis and mortality. We hypothesized that adiposity in the 2 years before diagnosis would be representative of the adiposity level at diagnosis because many men are diagnosed with localized prostate cancer that is usually asymptomatic (5,6). Subgroup analyses, such as by disease stage, tumor grade, or smoking status, by year of BMI measurement generally showed consistent positive associations. We found positive associations between BMI and mortality that were stronger in never smokers than in current and former smokers, consistent with studies that observed stronger associations for BMI and risk of advanced prostate cancer among never smokers (45-48).

Few studies to date have investigated the relationship between at diagnosis/postdiagnosis waist circumference (49-51) in patients with prostate cancer, but found no associations with mortality. In addition, thus far, no studies in patients with prostate cancer have investigated waist to hip ratio or hip circumference after diagnosis in relation to long-term survival outcomes. Our results indicated positive associations between waist circumference, hip circumference, waist to hip ratio, and mortality, although data were limited.

The biological mechanisms linking obesity to poor survival outcomes after prostate cancer diagnosis have not been fully elucidated, and additional research is required (52). Numerous metabolic imbalances and interrelated pathways, including altered sex steroid hormones, serum insulin levels, and free insulin-like growth factor 1 levels may influence prostate cancer prognosis (52-54). Obesity-induced hormone and inflammatory changes could facilitate tumor progression (37,55) as well as higher risk of metastasis (56-58) and death (37,59). Chronically elevated insulin levels could facilitate tumor progression (5,60) and development of comorbidities, including cardiovascular disease (61), a major cause of death in patients with prostate cancer (55). Obesity has been positively associated with higher neutrophil to lymphocyte ratio (62,63), and strong or highly suggestive evidence supports that high neutrophil to lymphocyte ratio is associated with worse survival outcomes in patients with prostate cancer (64). An elevated obesity-induced inflammatory environment could worsen treatment-related side effects, for example, leading to more severe or extended post-treatment cancer-related fatigue (65). Chronically elevated levels of glucose in blood can activate the insulin pathway and facilitate tumor progression, the repair of tumor cells after radiation therapy, or treatment resistance (65,66). Androgen-deprivation therapy is commonly given in fixed doses, irrespective of the body’s surface area, and this practice could result in insufficient testosterone suppression in men with obesity compared with men who have normal weight (67). Inadequate pharmacological castration has been associated with more aggressive tumor biology (68), higher risk of progression and metastasis, and higher rates of mortality after androgen-deprivation therapy (67,69). The influence of castration therapies at the molecular level needs to be better understood. Obesity-related gene transcription in the adipose tissue surrounding the prostate could trigger inflammatory and metabolic changes (eg, altered hormone homeostasis, altered tissue lipid composition) that could partially counteract the beneficial effects of castration therapies (70).

The strengths of this study include its prospective design, representation of men from 7 countries, detailed information about adiposity indices close to diagnosis, mortality outcomes, and confounders. Important sensitivity analyses were performed, such as by distinct subgroups of disease stage and tumor grade. The period after cancer diagnosis, during and after treatment, is particularly complex because it involves various biological, behavioral, and physiological changes (71). Involuntary lifestyle alterations related to the disease and its treatment could influence body composition and in turn negatively affect cancer outcomes (9,71-74). We did not have information about cancer treatment, complications, or disease recurrence. Adjusting for diagnosis year, disease stage, and tumor grade could have potentially mitigated the limitation of not having treatment information because these variables reflect, to some extent, the treatment received and improvements in available prostate cancer therapies over time (75-78). Lack of repeated postdiagnosis adiposity measurements did not enable us to examine how cumulative changes in adiposity (and other lifestyle factors) could influence mortality by performing time-varying analyses. Our analysis of prediagnosis or postdiagnosis adiposity combined, however, has (at least partly) accounted for this limitation. Some men did not have data on the confounders of our main model (mainly, disease stage and tumor grade), particularly for the postdiagnosis time frame. All methods to account for missing data have limitations, and we acknowledge that performing a complete-case analysis was rather simplistic but likely the best possible approach to deal with the missing data in this study. We have provided detailed information about data missingness for transparency. A missing-indicator analysis could lead to biased estimates (79), and multiple imputation could have been more useful, with smaller amounts of missing data across variables (32). We investigated potential selection bias, but no material differences were observed in important lifestyle and tumor characteristics of the men included in and excluded from the analyses because of missing data.

### Conclusions and future directions

Maintaining a healthy weight could lead to better prostate cancer prognosis. Additional well-designed and well-conducted observational and weight management intervention studies with larger sample sizes and repeated postdiagnosis measurements are needed. Mechanistic studies are essential to unravel the biological pathways driving tumor progression and mortality according to obesity-related factors and ultimately enable the design of targeted lifestyle interventions that will help men better cope with this disease.

## Supplementary Material

pkae070_Supplementary_Data

## Data Availability

For information about how to apply for gaining access to EPIC data or biospecimens, please follow the instructions at https://epic.iarc.fr/access.
