# Supplementary material for: Adiposity assessed close to diagnosis and prostate cancer prognosis in the EPIC study
Source: JNCI Cancer Spectr. 2024 Aug 24;8(5):pkae070. doi: 10.1093/jncics/pkae070 (PMC11410200; doi:10.1093/jncics/pkae070)

Adiposity assessed close to diagnosis and prostate cancer prognosis in the EPIC study.

Margarita Cariolou[1], MPH, Sofia Christakoudi[1], PhD, Marc J Gunter[1], PhD, Tim Key[2], DPhil, Aurora Pérez-Cornago[2], PhD, Ruth Travis[2], DPhil, Raul Zamora-Ros[3], PhD, Kristina Elin T Petersen[4], PhD, Anne Tjønneland[4,5], MD, PhD, Elisabete Weiderpass[6], MD, PhD, Rudolf Kaaks[7], PhD, Petra Seibold[7], PhD, Elif Inan-Eroglu[8], PhD, Matthias B Schulze[8,9], DrPH, Giovanna Masala[10], MD, Claudia Agnoli[11], MSc, Rosario Tumino[12], MD, Chiara Di Girolamo[13], PhD, Amaia Aizpurua[14,15], MSc, Miguel Rodriguez-Barranco[16,17,18], PhD, Carmen Santiuste[18,19], MD, Marcela Guevara[18,20,21], MD, PhD, Dagfinn Aune[1,22,23], PhD, Doris S M Chan[1], PhD, David C Muller[1], PhD, Konstantinos K Tsilidis, PhD [1,24¥]

Table of Contents

Supplementary Methods 3
Supplementary References 4
Supplementary Tables 5
Supplementary Table 1 – Relevant covariates based on subject matter knowledge. 5
Supplementary Table 2 – Total number of men and deaths in the models of each adiposity index. 7
Supplementary Table 3 – Categorical analysis according to the BMI (WHO categories) and all-cause mortality. 10
Supplementary Table 4 – Categorical analysis according to the BMI (WHO categories) and prostate cancer-specific mortality. 11
Supplementary Table 5 – Missing data from covariates included in the main models for each data collection timeframe. 12
Supplementary Table 6 – Comparison of major lifestyle and tumour characteristics of men with BMI data according to the eligibility criteria of the present study versus those excluded because they did not have BMI data and versus those with complete covariate data included in the main models. 13
Supplementary Table 7 – Demographic and tumour characteristics of the 1,968 men with prostate cancer, by BMI (WHO categories). 14
Supplementary Table 8 – Stratified analysis by smoking for those who have pre- or post-diagnosis BMI combined. 14
Supplementary Table 9 – Sensitivity analyses including individuals of Model 3, in Model 2 and Model 1 to explore influence of covariates. 15
Supplementary Figures 16
Supplementary Figure 1 – Total number of men with adiposity data according to the eligibility criteria of the present study and number of all-cause and prostate cancer-specific deaths. 16
Supplementary Figure 2 – Subgroup analysis by prostate cancer stage. 17
Supplementary Figure 3 – Subgroup analysis by prostate cancer grade. 18
Supplementary Figure 4 – Lag-analysis of BMI (excluding deaths in the first year of follow-up). 19
Supplementary Figure 5 – Analysis of post-diagnosis BMI adjusted for pre-diagnosis BMI. 20

Supplementary Figure 6 – Total number of men with BMI data at one and two years before diagnosis, and at each year post-diagnosis up to five years after. 21

Supplementary Figure 7 – Analysis of BMI for each year pre-diagnosis (up to two years pre-diagnosis). 22

Supplementary Figure 8 – Analysis of BMI for each year post-diagnosis (up to the third-year post-diagnosis). 23

Supplementary Figure 9 – Stratified analysis by smoking status for pre- or post-diagnosis BMI combined. 24

Supplementary Figure 10 – Additional adjustment for highest school level as proxy for socioeconomic status in the analysis of pre- or post-diagnosis BMI combined. 25

Supplementary Figure 11 – Analysis of waist circumference and all-cause and prostate cancer-specific mortality. 26

Supplementary Figure 12 – Analysis of hip circumference and all-cause and prostate cancer-specific mortality. 27

Supplementary Figure 13 – Analysis of waist-to-hip ratio and all-cause and prostate cancer-specific mortality. 28

Supplementary Methods

Study characteristics, eligibility criteria and anthropometric variable measurements – details.

Participants in EPIC, provided signed informed consent and were subsequently mailed lifestyle and diet questionnaires. Exceptions to the recruitment scheme: only women participated in the cohorts of Norway, Utrecht, Naples, and France; half of the Oxford cohort did not consume meat, or were fish eaters, lacto-ovo vegetarians or vegans; part of the Italian and Spanish cohorts were members of local blood donor associations; the Utrecht and Florence cohorts recruited women who participated in local breast cancer screening programmes. Weight was measured to the nearest 0.1 kg and height was measured dependent on the study centre – to the nearest 0.1, 0.5, or 1.0 cm with participants not wearing shoes. BMI was calculated as weight in kg divided by height in m^2. Waist circumference was measured either at the midpoint between the lower ribs and iliac crest or at the narrowest torso circumference.(1, 2)

Prostate cancer recurrence usually occurs within the first five years after initial treatment.(3-6) The post-diagnosis exposure window of the present study was therefore restricted to five years. Regarding the pre-diagnosis exposure window, adiposity status in the two years prior to diagnosis may be a good representation of the adiposity status at diagnosis, and likely not affected by the cancer itself since most prostate cancer tumours are diagnosed at early stages and are usually asymptomatic.(7, 8) Of the total, 7,763 men with prostate cancer 5,748 (74%) provided additional data in the follow-up questionnaire. From the 7,763 men, we further excluded 147 with a missing diagnosis date, 20 with a date of prostate cancer diagnosis that was after or the same as the date of death, three who had same date of diagnosis as end of follow-up/censoring, two with death date after end of follow-up and 118 from Greece since data was not available for this analysis. Total exclusions, N=290; eligible men with prostate cancer, N=7,473. One additional individual was excluded from the subset of men eligible for analysis, because the date of questionnaire/assessment was after the date of death or censoring, leaving 1,968 men with adiposity data either two years before or five years after diagnosis.

Identification of prostate cancer cases, follow up and outcome assessment.

Information on vital status, date and cause of death were ascertained through record linkages with population cancer registries, death indices and national health statistics in Denmark, Spain, The Netherlands, Italy, Sweden, and the UK. Active follow up of participants or their next-of kin or a combination of different sources of ascertainment including health insurance records, regional health departments, municipal registries, hospital records and pathology registries were used in Germany. The ICD-10 was used to define the causes of death. Outcomes of interest were all-cause and prostate cancer-specific mortality.

Definition of important covariates

The Cambridge physical activity index was derived by combining occupational activity level with recreational physical activity i.e., amount of time (hours/week) during the summer and winter spent cycling and in other exercise activities (jogging, swimming etc), and was defined as: inactive, moderately inactive, moderately active, active, unknown/missing.[(9)] In this study, tumour stage was defined using the Tumour–Node–Metastasis (TNM) code and if this information was not available, the EPIC stage classification was used as provided (i.e., localised or metastatic [“advanced”]): localised (T0-T2 and N0-NX and M0), advanced (T3-T4 and/or N1-N3 and/or M1), unknown/missing. Tumour grade was defined considering Gleason score and if this information was not available, the EPIC grade classification was used as provided: Gleason score 2-6 (well-differentiated), Gleason score 7 (moderately differentiated), Gleason score 8-10 (poorly/undifferentiated) or unknown/missing. Two variables were used for smoking, namely “smoking status”: never, former, current, unknown/missing and “lifetime cigarettes/day”.

Subgroup and sensitivity analyses for BMI

To assess bias due to reverse causation, the following analyses were performed: a) restricting to non-metastatic prostate cancer tumours at diagnosis, and b) excluding deaths that occurred in the first year of follow-up after the data collection/questionnaire (for the post-diagnosis and the pre- or post-diagnosis combined analysis). To check for potential selection bias, we compared important baseline lifestyle and tumour characteristics at diagnosis of the men with prostate cancer who had adiposity data according to our eligibility criteria (*N=1,968*) versus those who did not (*N=5,505*). We also compared the total men included in our study (*N=1,968*) to those included in the main analyses (excluding those who did not have data on covariates of the main model i.e., *N=942* for pre- or post-diagnosis BMI combined; *N=372* and *N=570* for post- and pre-diagnosis respectively).

Supplementary References

1. Haftenberger M, Lahmann PH, Panico S, *et al.* Overweight, obesity and fat distribution in 50- to 64-year-old participants in the European Prospective Investigation into Cancer and Nutrition (EPIC). *Public Health Nutr.* 2002;5(6b):1147-62.
2. Riboli E, Hunt KJ, Slimani N, *et al.* European Prospective Investigation into Cancer and Nutrition (EPIC): study populations and data collection. *Public Health Nutr.* 2002;5(6b):1113-24.
3. Trock BJ, Han M, Freedland SJ, *et al.* Prostate cancer-specific survival following salvage radiotherapy vs observation in men with biochemical recurrence after radical prostatectomy. *Jama.* 2008;299(23):2760-9.
4. Tourinho-Barbosa R, Srougi V, Nunes-Silva I, *et al.* Biochemical recurrence after radical prostatectomy: what does it mean? *Int Braz J Urol.* 2018;44(1):14-21.
5. Limonta P, M Moretti R, Marzagalli M, Montagnani Marelli M. Castration Resistant Prostate Cancer: From emerging molecular pathways to targeted therapeutic approaches. *Clinical Cancer Drugs.* 2014;1(1):11-27.
6. Chetta P, Zadra G. Metabolic reprogramming as an emerging mechanism of resistance to endocrine therapies in prostate cancer. *Cancer Drug Resist.* 2021;4(1):143-162.
7. Rawla P. Epidemiology of Prostate Cancer. *World J Oncol.* 2019;10(2):63-89.
8. Rebello RJ, Oing C, Knudsen KE, *et al.* Prostate cancer. *Nat Rev Dis Primers.* 2021;7(1):9.
9. Wareham NJ, Jakes RW, Rennie KL, *et al.* Validity and repeatability of a simple index derived from the short physical activity questionnaire used in the European Prospective Investigation into Cancer and Nutrition (EPIC) study. *Public Health Nutr.* 2003;6(4):407-13.

Supplementary Tables

Supplementary Table 1 – Relevant covariates based on subject matter knowledge.

Confounder	Relevance to obesity	Relevance to mortality (survival)
Age	Due to increased life expectancy obesity is more prevalent among older age groups. Ageing is associated with increased abdominal white adipose tissue and fat deposition in skeletal muscle.[1] Lifestyle changes in the elderly can result in excess fat tissue accumulation, that in turn accelerates the development of age-related diseases.[2]	Mortality increases with age (has shown to double every 6-7 years during adulthood).[3] Prostate cancer mortality rates strongly associated with age.[4]
Year of diagnosis	Obesity prevalence has doubled worldwide since 1980[5] and increased four-fold in men between 1975 and 2016[6] Projections indicate that obesity is expected to reach maximal levels for men between 2030 and 2052.[7]	Prostate cancer, detection, treatment, and survival outcomes have improved with time.[8-10]
Tumour stage	Aggressive prostate cancer has been positively associated with adiposity[5, 11-13] and inversely associated with localised prostate cancer[12, 14]	Survival rate/prognosis is strongly correlated to stage at diagnosis.[15] Five-year survival rate 90-99% for organ-confined PCa,[16] 30% for metastatic PCa[17] and poor prognosis of chemical castration resistant prostate cancer (9-30 months median survival).[18] Metastatic disease is the leading cause of deaths due to PCa.[19]
Gleason score/tumour grade	Positive association between obesity and high-grade prostate cancer tumours.[12, 20]	Gleason score is the main prognostic factor of prostate cancer progression and treatment.[15, 21] High grade tumours have poorer prognosis.[22]
Prostate-specific antigen (PSA) levels	Dilution of PSA levels due to obesity.[23, 24]	PSA value at diagnosis is one of the most important factors in risk stratification.[15, 25, 26]
Smoking	Quitting smoking has been associated with weight gain.[27] High tobacco consumption can lower body weight.[28]	Smoking increases risk of aggressive prostate cancer and prostate cancer-specific mortality.[29-31]
Physical activity	Increased physical activity associated with weight loss.[32]	Physical activity has been associated with lower risk of all-cause and prostate cancer-specific mortality.[33]
Socioeconomic status (education as proxy)	Socio-economic status is an important determinant of obesity development in adults.[34, 35]	Lower education and socio-economic status may increase risk of death in prostate cancer patients.[36]

References

1. Jura M, Kozak LP. Obesity and related consequences to ageing. *Age (Dordr)*. 2016;38(1):23.
2. Tchkonia T, Morbeck DE, Von Zglinicki T*, et al.* Fat tissue, aging, and cellular senescence. *Aging Cell*. 2010;9(5):667-84.
3. Ebeling M, Rau R, Malmström H*, et al.* The rate by which mortality increase with age is the same for those who experienced chronic disease as for the general population. *Age Ageing*. 2021;50(5):1633-40.
4. Rawla P. Epidemiology of Prostate Cancer. *World J Oncol*. 2019;10(2):63-89.
5. Fujita K, Hayashi T, Matsushita M*, et al.* Obesity, Inflammation, and Prostate Cancer. *J Clin Med*. 2019;8(2).
6. Sung H, Siegel RL, Torre LA*, et al.* Global patterns in excess body weight and the associated cancer burden. *CA Cancer J Clin*. 2019;69(2):88-112.
7. Janssen F, Bardoutsos A, Vidra N. Obesity Prevalence in the Long-Term Future in 18 European Countries and in the USA. *Obes Facts*. 2020;13(5):514-27.
8. Kim MM, Hoffman KE, Levy LB*, et al.* Improvement in prostate cancer survival over time: a 20-year analysis. *Cancer J*. 2012;18(1):1-8.
9. Barsouk A, Padala SA, Vakiti A*, et al.* Epidemiology, Staging and Management of Prostate Cancer. *Med Sci (Basel)*. 2020;8(3).
10. Nevedomskaya E, Baumgart SJ, Haendler B. Recent Advances in Prostate Cancer Treatment and Drug Discovery. *Int J Mol Sci*. 2018;19(5).
11. World Cancer Research Fund International/American Institute for Cancer Research Continuous Update Project Report: Diet, nutrition, physical activity, and prostate cancer 2018. Available from: https://www.wcrf.org/diet-and-cancer/cancer-types/].
12. Perez-Cornago A, Appleby PN, Pischon T*, et al.* Tall height and obesity are associated with an increased risk of aggressive prostate cancer: results from the EPIC cohort study. *BMC Med*. 2017;15(1):115.
13. Cao Y, Giovannucci E. Obesity and Prostate Cancer. *Recent Results Cancer Res*. 2016;208:137-53.
14. Discacciati A, Orsini N, Wolk A. Body mass index and incidence of localized and advanced prostate cancer--a dose-response meta-analysis of prospective studies. *Ann Oncol*. 2012;23(7):1665-71.
15. Pugliese D, Palermo G, Totaro A*, et al.* Clinical, pathological and molecular prognostic factors in prostate cancer decision-making process. *Urologia*. 2016;83(1):14-20.
16. Rebello RJ, Oing C, Knudsen KE*, et al.* Prostate cancer. *Nat Rev Dis Primers*. 2021;7(1):9.
17. Khan S, Chang SH, Hicks V*, et al.* Improved survival with post-diagnostic metformin and statin use in a racially diverse cohort of US Veterans with advanced prostate cancer. *Prostate Cancer Prostatic Dis*. 2021.
18. Xu MC, Huelster HL, Hatcher JB*, et al.* Obesity is Associated with Longer Survival Independent of Sarcopenia and Myosteatosis in Metastatic and/or Castrate-Resistant Prostate Cancer. *J Urol*. 2020.
19. Wang G, Zhao D, Spring DJ*, et al.* Genetics and biology of prostate cancer. *Genes Dev*. 2018;32(17-18):1105-40.
20. Genkinger JM, Wu K, Wang M*, et al.* Measures of body fatness and height in early and mid-to-late adulthood and prostate cancer: risk and mortality in The Pooling Project of Prospective Studies of Diet and Cancer. *Ann Oncol*. 2020;31(1):103-14.
21. Martin NE, Mucci LA, Loda M*, et al.* Prognostic determinants in prostate cancer. *Cancer J*. 2011;17(6):429-37.
22. Short E, Warren AY, Varma MJDH. Gleason grading of prostate cancer: a pragmatic approach. 2019;25(10):371-8.
23. Langlais CS, Cowan JE, Neuhaus J*, et al.* Obesity at Diagnosis and Prostate Cancer Prognosis and Recurrence Risk Following Primary Treatment by Radical Prostatectomy. *Cancer Epidemiol Biomarkers Prev*. 2019;28(11):1917-25.
24. Tomaszewski JJ, Chen YF, Bertolet M*, et al.* Obesity is not associated with aggressive pathologic features or biochemical recurrence after radical prostatectomy. *Urology*. 2013;81(5):992-6.

25. D'Amico AV, Chen MH, Roehl KA, *et al.* Preoperative PSA velocity and the risk of death from prostate cancer after radical prostatectomy. *N Engl J Med*. 2004;351(2):125-35.
26. Litwin MS, Tan HJ. The Diagnosis and Treatment of Prostate Cancer: A Review. *JAMA*. 2017;317(24):2532-42.
27. Tian J, Venn A, Otahal P, *et al.* The association between quitting smoking and weight gain: a systematic review and meta-analysis of prospective cohort studies. *Obes Rev*. 2015;16(10):883-901.
28. Winsløw UC, Rode L, Nordestgaard BG. High tobacco consumption lowers body weight: a Mendelian randomization study of the Copenhagen General Population Study. *Int J Epidemiol*. 2015;44(2):540-50.
29. Dayal HH, Polissar L, Dahlberg S. Race, socioeconomic status, and other prognostic factors for survival from prostate cancer. *J Natl Cancer Inst*. 1985;74(5):1001-6.
30. Kenfield SA, Stampfer MJ, Chan JM, *et al.* Smoking and prostate cancer survival and recurrence. *Jama*. 2011;305(24):2548-55.
31. Müezzinler A, Mons U, Gellert C, *et al.* Smoking and All-cause Mortality in Older Adults: Results From the CHANCES Consortium. *Am J Prev Med*. 2015;49(5):e53-e63.
32. Chin SH, Kahathuduwa CN, Binks M. Physical activity and obesity: what we know and what we need to know. *Obes Rev*. 2016;17(12):1226-44.
33. Friedenreich CM, Stone CR, Cheung WY, *et al.* Physical Activity and Mortality in Cancer Survivors: A Systematic Review and Meta-Analysis. *JNCI Cancer Spectr*. 2019;4(1):pkz080.
34. Sacks G, Swinburn B, Lawrence M. Obesity Policy Action framework and analysis grids for a comprehensive policy approach to reducing obesity. *Obes Rev*. 2009;10(1):76-86.
35. Masood B, Moorthy M. Causes of obesity: a review. *Clin Med (Lond)*. 2023;23(4):284-91.
36. DeRouen MC, Schupp CW, Koo J, *et al.* Impact of individual and neighborhood factors on disparities in prostate cancer survival. *Cancer Epidemiol*. 2018;53:1-11.

Supplementary Table 2 – Total number of men and deaths in the models of each adiposity index.

Total men	Total deaths (any death/all-causes)	Total deaths (prostate cancer-specific mortality)	Model number[a]
**BMI**			
**Pre- or post-diagnosis combined**			
1968	805	424	1 (no missing data)
982	326	163	2 (with Gleason 7)
835	275	134	2 (with Gleason 3+4/4+3)
**942**	**320**	**163**	**3 (with Gleason 7) – main model**
795	269	134	3 (with Gleason 3+4/4+3)
432	117	65	4 (with Gleason 7) – sensitivity analysis
374	108	58	4 (with Gleason 3+4/4+3) – sensitivity analysis
**Post-diagnosis**			
968	406	209	1 (no missing data)
394	128	63	2 (with Gleason 7)
344	110	52	2 (with Gleason 3+4/4+3)
**372**	**126**	**63**	**3 (with Gleason 7) – main model**
322	108	52	3 (with Gleason 3+4/4+3)
142	35	15	4 (with Gleason 7) – sensitivity analysis
121	30	11	4 (with Gleason 3+4/4+3) – sensitivity analysis
**Pre-diagnosis**			
1000	399	215	1 (no missing data)
588	198	100	2 (with Gleason 7)
491	174	82	2 (with Gleason 3+4/4+3)
**570**	**194**	**100**	**3 (with Gleason 7) – main model**
473	161	82	3 (with Gleason 3+4/4+3)
290	82	50	4 (with Gleason 7) – sensitivity analysis
253	78	47	4 (with Gleason 3+4/4+3) – sensitivity analysis
**Waist circumference**			
**Pre- or post-diagnosis combined**			
1091	467	272	1 (no missing data)
376	124	79	2 (with Gleason 7)
310	105	69	2 (with Gleason 3+4/4+3)
**362**	**120**	**79**	**3 (with Gleason 7) – main model**
296	101	69	3 (with Gleason 3+4/4+3)
174	62	43	4 (with Gleason 7) – sensitivity analysis
141	56	38	4 (with Gleason 3+4/4+3) – sensitivity analysis
**Post-diagnosis**			
544	240	137	1 (no missing data)
124	35	26	2 (with Gleason 7)
109	31	23	2 (with Gleason 3+4/4+3)
**117**	**34**	**26**	**3 (with Gleason 7) – main model**
102	30	23	3 (with Gleason 3+4/4+3)
38	10	8	4 (with Gleason 7) – sensitivity analysis
31	8	6	4 (with Gleason 3+4/4+3) – sensitivity analysis
**Pre-diagnosis**			
547	227	135	1 (no missing data)
252	89	53	2 (with Gleason 7)
201	74	46	2 (with Gleason 3+4/4+3)
**245**	**86**	**53**	**3 (with Gleason 7) – main model**
194	71	46	3 (with Gleason 3+4/4+3)
136	52	35	4 (with Gleason 7) – sensitivity analysis
110	48	33	4 (with Gleason 3+4/4+3) – sensitivity analysis
**Hip circumference**			
**Pre- or post-diagnosis combined**			
793	348	191	1 (no missing data)
179	48	23	2 (with Gleason 7)

129	36	19	2 (with Gleason 3+4/4+3)
**167**	**45**	**23**	**3 (with Gleason 7) – main model**
117	33	19	3 (with Gleason 3+4/4+3)
75	22	14	4 (with Gleason 7) – sensitivity analysis
51	19	12	4 (with Gleason 3+4/4+3) – sensitivity analysis
**Post-diagnosis**			
403	181	97	1 (no missing data)
46	4	2	2 (with Gleason 7)
39	4	2	2 (with Gleason 3+4/4+3)
**39**	**3**	**2**	**3 (with Gleason 7) – main model**
32	3	2	3 (with Gleason 3+4/4+3)
8	1	0	4 (with Gleason 7) – sensitivity analysis
6	1	0	4 (with Gleason 3+4/4+3) – sensitivity analysis
**Pre-diagnosis**			
390	167	94	1 (no missing data)
133	44	21	2 (with Gleason 7)
90	32	17	2 (with Gleason 3+4/4+3)
**128**	**42**	**21**	**3 (with Gleason 7) – main model**
85	30	17	3 (with Gleason 3+4/4+3)
67	21	14	4 (with Gleason 7) – sensitivity analysis
45	18	12	4 (with Gleason 3+4/4+3) – sensitivity analysis
**Waist-to-hip ratio**			
Pre- or post-diagnosis combined			
793	348	191	1 (no missing data)
179	48	23	2 (with Gleason 7)
129	36	19	2 (with Gleason 3+4/4+3)
**167**	**45**	**23**	**3 (with Gleason 7) – main model**
117	33	19	3 (with Gleason 3+4/4+3)
75	22	14	4 (with Gleason 7) – sensitivity analysis
51	19	12	4 (with Gleason 3+4/4+3) – sensitivity analysis
**Post-diagnosis**			
403	181	97	1 (no missing data)
46	4	2	2 (with Gleason 7)
39	4	2	2 (with Gleason 3+4/4+3)
**39**	**3**	**2**	**3 (with Gleason 7) – main model**
32	3	2	3 (with Gleason 3+4/4+3)
8	1	0	4 (with Gleason 7) – sensitivity analysis
6	1	0	4 (with Gleason 3+4/4+3) – sensitivity analysis
**Pre-diagnosis**			
390	167	94	1 (no missing data)
133	44	21	2 (with Gleason 7)
90	32	17	2 (with Gleason 3+4/4+3)
**128**	**42**	**21**	**3 (with Gleason 7) – main model**
85	30	17	3 (with Gleason 3+4/4+3)
67	21	14	4 (with Gleason 7) – sensitivity analysis
45	18	12	4 (with Gleason 3+4/4+3) – sensitivity analysis

[a]*Model 1* (*minimally adjusted*), adjusted for age (continuous) and year of diagnosis (continuous) – no missing data.
Model 2 additionally adjusted for stage (categorical) and grade (categorical) – missing data eliminated.
Model 3 (*main model utilised in the present study*) additionally adjusted for smoking status (categorical) – missing data eliminated. *Model 4* (as sensitivity analysis), adjusted additionally for number of cigarettes/days (continuous) + physical activity (categorical) + log-transformed PSA levels (continuous) – missing data eliminated.
Survival time was calculated in days as difference between the date of death, emigration, withdrawal/lost to follow-up or last follow-up, whichever occurred first and date of either the at recruitment or follow-up anthropometric assessment/questionnaire.

Note: Gleason score 7 tumours have significant clinical heterogeneity[1] and different prognosis depending on the primary and secondary tumour pattern.[2,3] Previous studies found that prostate cancer patients with Gleason 4+3 tumours had higher risk of prostate cancer-specific mortality than those with Gleason 3+4 tumours.[4] Additional analyses with Gleason 7 tumours in two distinct categories i.e., 7:3+4 and 7:4+3 resulted in similar output to the analyses with the Gleason 7 tumours as a single group, therefore we present results for Gleason score 7 as a single group.

References:

1. Pugliese D, Palermo G, Totaro A, *et al.* Clinical, pathological and molecular prognostic factors in prostate cancer decision-making process. *Urologia*. 2016;83(1):14-20.
2. Short E, Warren AY, Varma MJDH. Gleason grading of prostate cancer: a pragmatic approach. 2019;25(10):371-8.
3. Epstein JI, Zelefsky MJ, Sjoberg DD, *et al.* A Contemporary Prostate Cancer Grading System: A Validated Alternative to the Gleason Score. *Eur Urol*. 2016;69(3):428-35.
4. Wright JL, Salinas CA, Lin DW, *et al.* Prostate cancer specific mortality and Gleason 7 disease differences in prostate cancer outcomes between cases with Gleason 4 + 3 and Gleason 3 + 4 tumors in a population based cohort. *J Urol*. 2009;182(6):2702-7.

Supplementary Table 3 – Categorical analysis according to the BMI (WHO categories) and all-cause mortality.						
	Pre- or post-diagnosis BMI		Post-diagnosis BMI		Pre-diagnosis BMI	
	N_e/N_t	HR[a] (95% CI)	N_e/N_t	HR[a] (95% CI)	N_e/N_t	HR[a] (95% CI)
Age at diagnosis	320/942	1.06 (1.04-1.09)	126/372	1.07 (1.03-1.10)	194/570	1.07 (1.04-1.10)
Year of diagnosis	320/942	0.90 (0.85-0.95)	126/372	0.80 (0.69-0.93)	194/570	0.94 (0.87-1.00)
BMI (WHO categories)						
Underweight (<18.5 kg/m^2)	4/5	0.91 (0.32-2.58)	0/0	-	4/5	0.91 (0.30-2.73)
Normal weight (18.5-24.9 kg/m^2)	99/307	1	44/135	1	55/172	1
Overweight (25-29.9 kg/m^2)	163/493	1.24 (0.95-1.61)	67/190	1.12 (0.75-1.68)	96/303	1.28 (0.90-1.81)
Obese (>=30 kg/m^2)	54/137	1.87 (1.32-2.66)	15/47	1.52 (0.81-2.83)	39/90	2.08 (1.34-3.24)
Tumour stage according to the TNM code or/otherwise the EPIC grade classification (as provided; coded as localised or advanced).						
Localised (T0-T2 and N0-NX and M0 otherwise the EPIC grade classification “localised”)	175/656	1	80/267	1	95/389	1
Advanced (T3-T4 and/or N1-N3 and/or M1 otherwise the EPIC grade classification “metastatic”)	145/286	1.95 (1.52-2.50)	46/105	1.38 (0.91-2.08)	99/181	2.45 (1.77-3.37)
Tumour grade according to Gleason score or the EPIC grading variable						
Gleason score 2-6 (or EPIC grade classification “well-differentiated”)	106/427	1	51/190	1	55/237	1
Gleason score 7 (or EPIC grade classification “moderately differentiated”)	118/359	1.23 (0.92-1.64)	41/123	1.25 (0.80-1.96)	77/236	1.36 (0.92-2.00)
Gleason score 8-10 (or EPIC grade classification “poorly/undifferentiated”)	96/156	2.67 (1.95-3.64)	34/59	2.51 (1.54-4.09)	62/97	2.97 (1.95-4.52)
Smoking status						
Never smoker	97/334	1	36/124	1	61/210	1
Former smoker	149/473	1.03 (0.79-1.35)	65/205	1.03 (0.68-1.58)	84/268	0.99 (1.01-1.40)
Current smoker	74/135	2.35 (1.71-3.23)	25/43	3.08 (1.78-5.32)	49/92	2.21 (1.48-3.31)

Abbreviations: N_e=Number of events; N_t=Total number of men; EPIC=European Prospective Investigation into Cancer and Nutrition; WHO=World Health Organisation
[a]Model adjusted for: age of diagnosis, year of diagnosis, tumour stage, tumour grade, smoking status and stratified by EPIC country.
Empty cells (-): No data available.

**Supplementary Table 4 – Categorical analysis according to the BMI (WHO categories) and prostate cancer-specific mortality.**						
	Pre- or post-diagnosis BMI		Post-diagnosis BMI		Pre-diagnosis BMI	
	N_e/N_t	HR[a] (95% CI)	N_e/N_t	HR[a] (95% CI)	N_e/N_t	HR[a] (95% CI)
Age at diagnosis	163/942	1.03 (1.00-1.06)	63/372	1.04 (0.99-1.08)	100/570	1.03 (0.99-1.07)
Year of diagnosis	163/942	0.91 (0.84-0.98)	63/372	0.87 (0.70-1.07)	100/570	0.93 (0.86-1.04)
BMI (WHO categories)						
Underweight (<18.5 kg/m^2)	0/5	-	0/0	-	0/5	-
Normal weight (18.5-24.9 kg/m^2)	49/307	1	17/135	1	32/172	1
Overweight (25-29.9 kg/m^2)	87/493	1.50 (1.04-2.17)	39/190	1.81 (0.98-3.34)	48/303	1.41 (0.87-2.28)
Obese (>=30 kg/m^2)	27/137	2.05 (1.24-3.38)	7/47	2.12 (0.82-5.51)	20/90	2.08 (1.13-3.84)
Tumour stage according to the TNM code otherwise the EPIC grade classification (as provided; coded as localised or advanced).						
Localised (T0-T2 and N0-NX and M0 otherwise the EPIC grade classification “localised”)	61/656	1	32/267	1	29/389	1
Advanced (T3-T4 and/or N1-N3 and/or M1 otherwise the EPIC grade classification “metastatic”)	102/286	3.78 (2.65-5.39)	31/105	1.82 (1.03-3.22)	71/181	5.89 (3.63-9.57)
Tumour grade according to Gleason score or the EPIC grading variable						
Gleason score 2-6 (or EPIC grade classification “well-differentiated”)	34/427	1	17/190	1	17/237	1
Gleason score 7 (or EPIC grade classification “moderately differentiated”)	59/359	1.73 (1.09-2.74)	22/123	2.07 (1.03-4.15)	37/236	1.80 (0.95-3.39)
Gleason score 8-10 (or EPIC grade classification “poorly/undifferentiated”)	70/156	4.58 (2.90-7.22)	24/59	4.63 (2.29-9.34)	46/97	5.06 (2.69-9.52)
Smoking status						
Never smoker	56/334	1	21/124	1	35/210	1
Former smoker	68/473	0.85 (0.59-1.23)	29/205	0.67 (0.37-1.22)	39/268	0.95 (0.58-1.55)
Current smoker	39/135	1.80 (1.16-2.75)	13/43	2.13 (1.01-4.49)	26/92	1.77 (1.03-3.05)

Abbreviations: N_e=Number of events; N_t=Total number of men; TNM= Tumour–Node–Metastasis; EPIC=European Prospective Investigation into Cancer and Nutrition; WHO=World Health Organisation

[a]Model adjusted for: age of diagnosis, year of diagnosis, tumour stage, tumour grade, smoking status and stratified by EPIC country.

Empty cells (-): No data available.

Supplementary Table 5 – Missing data from covariates included in the main models for each data collection timeframe.

% data missing[a]	Pre-diagnosis				Post-diagnosis				Pre- or post-diagnosis (combined)			
	**BMI** n=1000	**WC** n=547	**HC** n=390	**WHR** n=390	**BMI** n=968	**WC** n=544	**HC** n=403	**WHR** n=403	**BMI** n=1968	**WC** n=1091	**HC** n=793	**WHR** n=793
**Covariate**												
PSA	33%	40%	55%	55%	56%	68%	89%	89%	45%	54%	72%	72%
Stage (TNM code if available and if not the EPIC classification)	29%	39%	48%	48%	49%	69%	88%	88%	39%	54%	68%	68%
Grade (Gleason score if available and if not the EPIC classification)	25%	32%	40%	40%	43%	58%	67%	67%	34%	45%	54%	54%
Lifetime number of cigarettes/day	29%	28%	21%	21%	33%	33%	26%	26%	31%	30%	23%	23%
Physical activity	16%	3%	3%	3%	15%	3%	4%	4%	15%	3%	4%	4%
Smoking status	2%	2%	3%	3%	5%	5%	6%	6%	4%	3%	4%	4%

[a]Year of diagnosis and age of diagnosis: no missing data.
The % of missing data are presented for each adiposity variable in the following order: *Body mass index (BMI), waist circumference (WC), hip circumference (HC) and waist-to-hip ratio (WHR).* The % of missing data for hip circumference and waist-to-hip ratio was the same.
Abbreviations: BMI, Body mass index; WC, waist circumference; Hip circumference, HC; WHR, waist-to-hip ratio; PSA, Prostate specific antigen; TNM, Tumour–Node–Metastasis; EPIC, European Prospective Investigation into Cancer and Nutrition.

Supplementary Table 6 – Comparison of major lifestyle and tumour characteristics of men with BMI data according to the eligibility criteria of the present study versus those excluded because they did not have BMI data and versus those with complete covariate data included in the main models.

	All men with prostate cancer and BMI data according to the eligibility criteria (before exclusions due to missing covariate data)	Men excluded from the total eligible sample of men with prostate cancer in EPIC. (*N=7,473*) if they did not meet eligibility criteria for this analysis (i.e., no BMI data close to diagnosis - two years before or five years after)	Men with prostate cancer who had complete data. (i.e., included in the main BMI model that was adjusted for age and year of diagnosis, tumour stage and grade and smoking status)		
	N=1,968	*N=5,505*	*N=942* (pre/post diagnosis)	*N=372* (post-diagnosis)	*N=570* (pre-diagnosis)
**Age at diagnosis, years, median (p5-p95)**	66 (55-77)	69 (58-80)	66 (56-76)	65 (56-76)	66 (56-77)
**Stage of tumour (based on the EPIC stage classification)**					
In situ, n (%)	0 (0)	0 (0)	0 (0)	0 (0)	0 (0)
Localised, n (%)	710 (36)	1525 (27)	570 (56)	226 (56)	344 (56)
Metastatic, n (%)	49 (3)	101 (2)	27 (3)	13 (3)	14 (2)
Metastatic regional, n (%)	77 (4)	90 (2)	67 (7)	30 (7)	37 (6)
Metastatic distant, n (%)	56 (3)	40 (1)	39 (4)	13 (3)	26 (4)
Unknown/missing, n (%)	1076 (55)	3749 (68)	239 (25)	90 (24)	149 (26)
**Stage of the tumour – based on the TNM code and if not available, the EPIC stage classification variable.**					
Localised (T0-T2 and N0-NX and M0), n (%)	824 (42)	1866 (34)	656 (70)	267 (72)	389 (68)
Advanced (T3-T4 and/or N1-N3 and/or M1), n (%)	382 (19)	1024 (19)	286 (30)	105 (28)	181 (32)
Unknown/missing, n (%)	762 (39)	2615 (48)	-	-	-
**Stage of the tumour - considering the TNM code and if not available the EPIC stage classification variable – excluding the missing category from the total.**					
Localised (T0-T2 and N0-NX and M0), n (%)	824 (68)	1866 (65)	656 (70)	267 (72)	389 (68)
Advanced (T3-T4 and/or N1-N3 and/or M1), n (%)	382 (32)	1024 (35)	286 (30)	105 (28)	181 (32)
**Grading of the tumour (based on the EPIC classification)**					
Well differentiated, n (%)	58 (3)	176 (3)	42 (4)	17 (5)	25 (4)
Moderately differentiated, n (%)	282 (14)	512 (9)	210 (22)	92 (25)	118 (21)
Poor/undifferentiated, n (%)	91 (5)	255 (5)	72 (8)	21 (6)	51 (9)
Unknown/missing, n (%)	1537 (78)	4562 (83)	618 (66)	242 (65)	376 (66)
**Grading of the tumour – based on the Gleason score and if not available on the EPIC classification.**					
**Gleason score 7 (moderately differentiated) as a separate category**					
Gleason score 2-6 (well-differentiated), n (%)	632 (32)	1418 (26)	427 (45)	190 (51)	237 (42)
Gleason score 7 (moderately differentiated), n (%)	456 (23)	1231 (22)	359 (38)	123 (33)	236 (41)
Gleason score 8-10 (poorly or undifferentiated), n (%)	211 (11)	641 (12)	156 (17)	59 (16)	97 (17)
Unknown/undetermined, n (%)	669 (34)	2215 (40)	-	-	-
**Gleason score 7 (moderately differentiated) as a separate category (also considering the EPIC grading variable) – excluding the missing category from the total.**					
Gleason score 2-6 (well-differentiated), n (%)	632 (49)	1418 (43)	427 (45)	190 (51)	237 (42)
Gleason score 7 (moderately differentiated), n (%)	456 (35)	1231 (37)	359 (38)	123 (33)	236 (41)
Gleason score 8-10 (poorly or undifferentiated), n (%)	211 (16)	641 (19)	156 (17)	59 (16)	97 (17)
**Anthropometry (BMI baseline/at recruitment)**					
BMI kg/m^2, median (P5–P95)	26 (21-33)	26 (21-32)	26 (22-33)	26 (21-31)	26 (22-33)
Underweight (<18.5 kg/m^2), n (%)	8 (0)	19 (0)	4 (0)	0 (0)	4 (1)
Normal weight (18.5-24.9 kg/m^2), n (%)	676 (35)	1942 (35)	322 (34)	139 (37)	183 (32)
Overweight (25-29.9 kg/m^2), n (%)	1025 (52)	2824 (51)	488 (52)	191 (51)	297 (52)
Obese (≥30 kg/m^2), n (%)	259 (13)	720 (13)	128 (14)	42 (11)	86 (15)
Unknown/missing, n (%)	-	-	-	-	-
**Smoking status (at baseline/recruitment)**					
Never, n (%)	640 (33)	1884 (34)	325 (35)	124 (33)	201 (35)
Former, n (%)	928 (47)	2133 (39)	416 (44)	166 (45)	250 (44)
Current, n (%)	378 (19)	1418 (26)	193 (20)	77 (21)	116 (20)
Unknown/missing, n (%)	22 (1)	70 (1)	8 (1)	5 (1)	3 (1)
**Cambridge physical activity index (at baseline/recruitment)**					
Inactive, n (%)	479 (24)	1085 (20)	217 (23)	87 (23)	130 (23)
Moderately inactive, n (%)	668 (34)	1757 (32)	307 (33)	110 (30)	197 (35)
Moderately active, n (%)	427 (22)	1299 (24)	227 (24)	99 (27)	128 (22)
Active, n (%)	356 (18)	1272 (23)	176 (19)	69 (19)	107 (19)
Unknown/missing, n (%)	38 (2)	92 (2)	15 (2)	7 (2)	8 (1)
**Log PSA Levels, median (p5-p95)**	2.4 (1.1-4.9)	2.3 (1.4-5.4)	2.4 (1.1-5.0)	2.3 (1.2-4.8)	2.5 (1.1-5.3)

Note: %s rounded to the nearest whole number.
Abbreviations: BMI, Body mass index; PSA, Prostate specific antigen; TNM, Tumour–Node–Metastasis; EPIC, European Prospective Investigation into Cancer and Nutrition.
Empty cells (-): Unknown/missing data.

Supplementary Table 7 – Demographic and tumour characteristics of the 1,968 men with prostate cancer, by BMI (WHO categories).

	Underweight (BMI<18.5) (*N=8*)	Normal weight (BMI: 18.5-24.9) (*N=643*)	Overweight (BMI: 25-29.9) (*N=1,037*)	Obese (BMI≥30) (*N=280*)
**Follow-up time in study (time from return of either baseline or follow-up questionnaire until censoring) median years) (p5-p95)**	6.7 (1.6-13.6)	9.7 (1.8-18.1)	9.7 (2.2-17.5)	8.4 (2.2-16.7)
**Age at diagnosis, years, median (p5-p95)**	69 (60-78)	66 (55-78)	66 (56-77)	65 (55-77)
**Stage of tumour considering the TNM code or the EPIC stage classification**				
Localised (T0-T2 and N0-NX and M0), n (%)	2 (25)	279 (43)	434 (42)	109 (39)
Advanced (T3-T4 and/or N1-N3 and/or M1), n (%)	3 (38)	116 (18)	201 (19)	62 (22)
Unknown/missing, % (n)	3 (38)	248 (39)	402 (38)	109 (39)
**Grading of the tumour**				
Well differentiated, n (%)	0 (0)	10 (2)	30 (3)	18 (6)
Moderately differentiated, n (%)	0 (0)	76 (12)	155 (15)	51 (18)
Poor/undifferentiated, n (%)	0 (0)	26 (4)	51 (5)	14 (5)
Unknown/missing, n (%)	8 (100)	531 (83)	801 (77)	197 (70)
**Gleason score 7 (moderately differentiated) as a separate category (also considering the EPIC grading variable)**				
Gleason score 2-6 (well-differentiated), n (%)	3 (38)	198 (31)	327 (32)	104 (37)
Gleason score 7 (moderately differentiated), n (%)	2 (25)	152 (24)	247 (24)	55 (20)
Gleason score 8-10 (poorly or undifferentiated), n (%)	0 (0)	68 (11)	116 (11)	27 (10)
Unknown/missing, n (%)	3 (38)	225 (35)	347 (33)	94 (34)
**Lifestyle characteristics**				
**Smoking status**				
Never, n (%)	2 (25)	241 (37)	321 (31)	81 (29)
Former, n (%)	2 (25)	274 (43)	551 (53)	152 (54)
Current, n (%)	4 (50)	105 (16)	125 (12)	36 (13)
Unknown/missing, n (%)	0 (0)	23 (4)	40 (4)	11 (4)
**Cambridge physical activity index**				
Inactive, n (%)	3 (38)	138 (21)	268 (26)	86 (31)
Moderately inactive, n (%)	1 (13)	176 (13)	335 (32)	79 (28)
Moderately active, n (%)	0 (0)	135 (21)	149 (14)	38 (14)
Active, n (%)	2 (25)	84 (13)	137 (13)	33 (12)
Unknown/missing, n (%)	2 (25)	110 (17)	148 (14)	44 (16)
**PSA Levels, median (p5-p95)**	17 (12-29)	11 (4-288)	10 (3-104)	10 (2-186)

Abbreviations: BMI=Body mass index; PSA, Prostate specific antigen; TNM, Tumour–Node–Metastasis; EPIC, European Prospective Investigation into Cancer and Nutrition.

Supplementary Table 8 – Stratified analysis by smoking for those who have pre- or post-diagnosis BMI combined.

	N_e/N_t	Never smokers	N_e/N_t	Current smokers	N_e/N_t	Former smokers	*p-interaction*
**BMI (per 5 kg/m²)**		**HR[a] (95% CI)**		**HR[a] (95% CI)**		**HR[a] (95% CI)**	
**All-cause mortality**	97/334	1.62 (1.24-2.14)	74/135	1.45 (1.07-1.98)	149/473	0.98 (0.76-1.28)	
p-nonlinearity		0.63		0.28		0.53	
							0.01
**Prostate cancer-specific mortality**	56/334	1.92 (1.37-2.69)	39/135	1.30 (0.86-1.98)	68/473	1.12 (0.78-1.63)	
p-nonlinearity		0.34		0.02		0.89	
							0.07

[a]Main model (model 3): adjusted for age, year of diagnosis, stage, grade and stratified by EPIC country.
Abbreviations: HR, Hazard Ratio; CI, Confidence Interval; N_e, number of events; N_t, total number of men with prostate cancer. BMI=Body mass index; EPIC, European Prospective Investigation into Cancer and Nutrition.

**Supplementary Table 9 – Sensitivity analyses including individuals of Model 3, in Model 2 and Model 1 to explore influence of covariates.**			
	N_e/N_t	**HR[a] (95% CI)**	**HR[b] (95% CI)**
**BMI (per 5 kg/m^2)**			
All-cause mortality:			
Pre- or post-diagnosis combined	320/942	1.22 (1.03-1.43)	1.22 (1.04-1.43)
Pre-diagnosis	194/570	1.34 (1.10-1.64)	1.29 (1.06-1.57)
Post-diagnosis	126/372	1.02 (0.77-1.36)	1.07 (0.80-1.42)
Prostate cancer-specific mortality:			
Pre- or post-diagnosis combined	163/942	1.36 (1.10-1.69)	1.39 (1.12-1.72)
Pre-diagnosis	100/570	1.43 (1.10-1.86)	1.46 (1.12-1.90)
Post-diagnosis	63/372	1.26 (0.86-1.84)	1.36 (0.92-2.02)
**Waist circumference (per 10 cm)**			
All-cause mortality:			
Pre- or post-diagnosis combined	120/362	1.05 (0.87-1.26)	1.10 (0.92-1.31)
Pre-diagnosis	86/245	1.15 (0.93-1.42)	1.26 (1.03-1.54)
Post-diagnosis	34/117	0.83 (0.57-1.23)	0.72 (0.46-1.13)
Prostate cancer-specific mortality:			
Pre- or post-diagnosis combined	79/362	1.13 (0.90-1.41)	1.21 (0.98-1.51)
Pre-diagnosis	53/245	1.26 (0.96-1.66)	1.45 (1.13-1.86)
Post-diagnosis	26/117	0.91 (0.59-1.40)	0.80 (0.48-1.35)
**Hip circumference (per 10 cm)**			
All-cause mortality:			
Pre- or post-diagnosis combined	45/167	0.82 (0.53-1.27)	1.05 (0.68-1.63)
Pre-diagnosis	42/128	0.89 (0.56-1.41)	1.15 (0.72-1.84)
Post-diagnosis	3/39	(Limited data)	(Limited data)
Prostate cancer-specific mortality:			
Pre- or post-diagnosis combined	23/167	1.04 (0.57-1.89)	1.46 (0.81-2.62)
Pre-diagnosis	21/128	1.19 (0.62-2.30)	1.91 (0.97-3.78)
Post-diagnosis	2/39	(Limited data)	(Limited data)
**Waist-to-hip ratio (per 0.1 unit)**			
All-cause mortality:			
Pre- or post-diagnosis combined	45/167	1.16 (0.73-1.84)	1.23 (0.74-2.05)
Pre-diagnosis	42/128	1.16 (0.70-1.92)	1.29 (0.74-2.24)
Post-diagnosis	3/55	(Limited data)	(Limited data)
Prostate cancer-specific mortality:			
Pre- or post-diagnosis combined	23/167	1.41 (0.75-2.67)	1.74 (0.81-3.76)
Pre-diagnosis	21/128	1.36 (0.67-2.76)	2.00 (0.83-4.85)
Post-diagnosis	2/39	(Limited data)	(Limited data)

[a]Model with the same participants as in main model (model 3) adjusted for age and year of diagnosis and stratified by EPIC country.
[b]Model with the same participants as in main model (model 3) adjusted for age and year of diagnosis, stage, and grade and stratified by EPIC country.
Note: The main model (model 3) was adjusted for age, year of diagnosis, stage, grade, smoking status and stratified by EPIC country.
Abbreviations: HR, Hazard Ratio; CI, Confidence Interval; N_e, number of events; N_t, total number of men with prostate cancer; BMI=Body mass index.

Supplementary Figures

Supplementary Figure 1 – Total number of men with adiposity data according to the eligibility criteria of the present study and number of all-cause and prostate cancer-specific deaths.
Diagrams present the data before exclusions of missing data in the pre-diagnosis, post-diagnosis, and pre/post diagnosis combined analysis for **(A)** BMI; **(B)** waist circumference; **(C)** hip circumference; **(D)** waist-to-hip ratio.

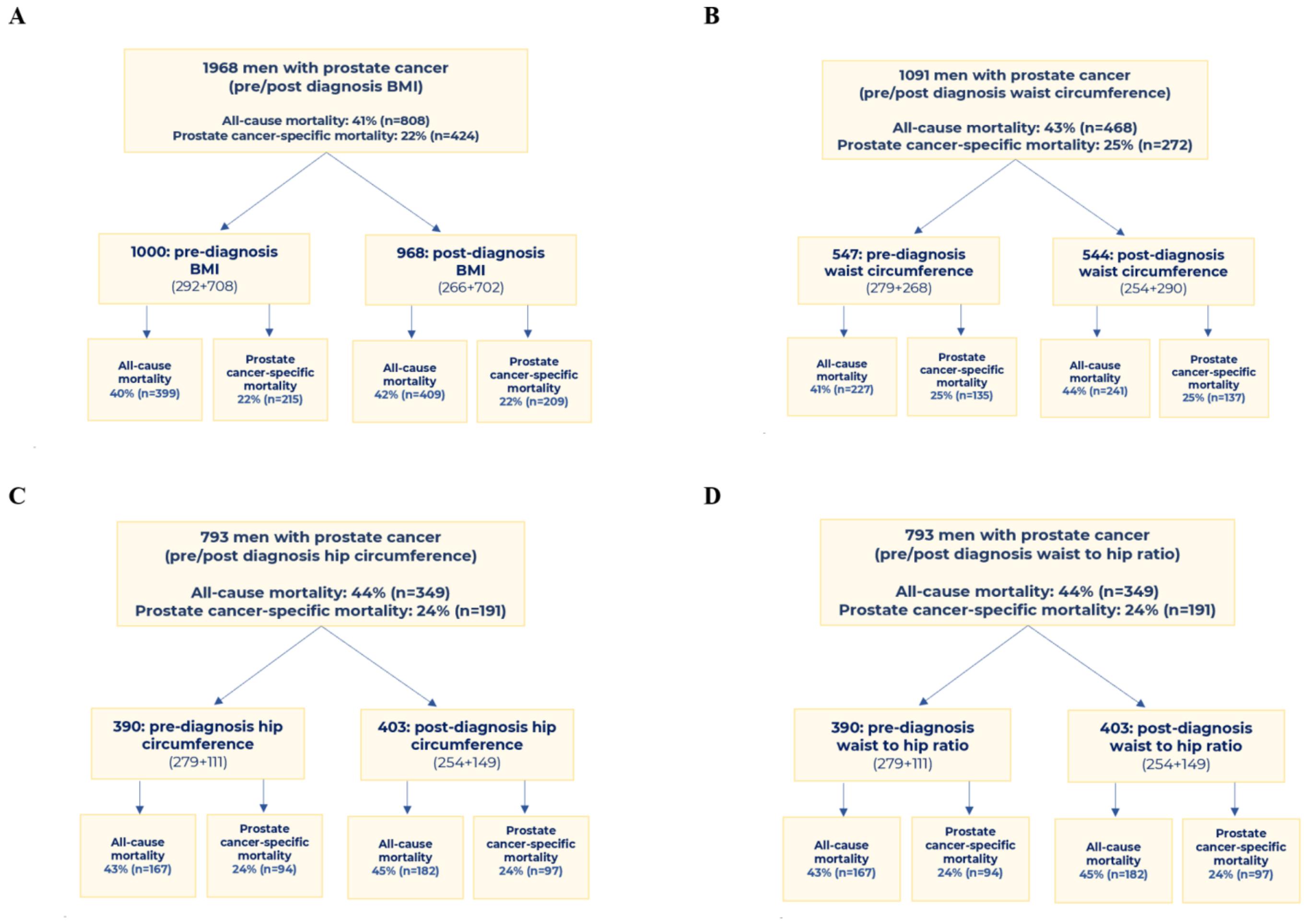

Supplementary Figure 2 – Subgroup analysis by prostate cancer stage.

Hazard ratios (HRs) from restricted cubic spline analysis, describing the association between BMI (kg/m^2) collected pre- or post-diagnosis combined in men with localised prostate cancer and A) all-cause mortality (men/deaths=656/175) and B) prostate cancer-specific mortality (men/deaths=656/61); in men with advanced prostate cancer and C) all-cause mortality (men/deaths=286/145) and D) prostate cancer-specific mortality (men/deaths=286/102); in those with metastatic prostate cancer and E) all-cause mortality (men/deaths=123/87) F) prostate cancer-specific mortality (men/deaths=123/78); excluding those with metastatic prostate cancer and G) all-cause mortality (men/deaths=819/233) and H) prostate cancer-specific mortality (men/deaths=819/85); HRs are based on the main Cox proportional hazards regression model of the present study adjusted for age (continuous), year of diagnosis (continuous), tumour grade (categorical) and smoking status (categorical) and stratified by EPIC country. The model excluding those with metastatic prostate cancer was also adjusted for stage. Knots at the 10th, 50th and 90th percentiles of BMI. Median BMI of the individuals included in analyses was used as referent: 26.2 kg/m^2 for those with localised prostate cancer, 26.3 kg/m^2 for those with advanced prostate cancer, 26.2 kg/m^2 in the analysis of men without metastasis and 26.2 kg/m^2 in men with metastasis only. The smooth density plot represents the density of the population across the spline variable.

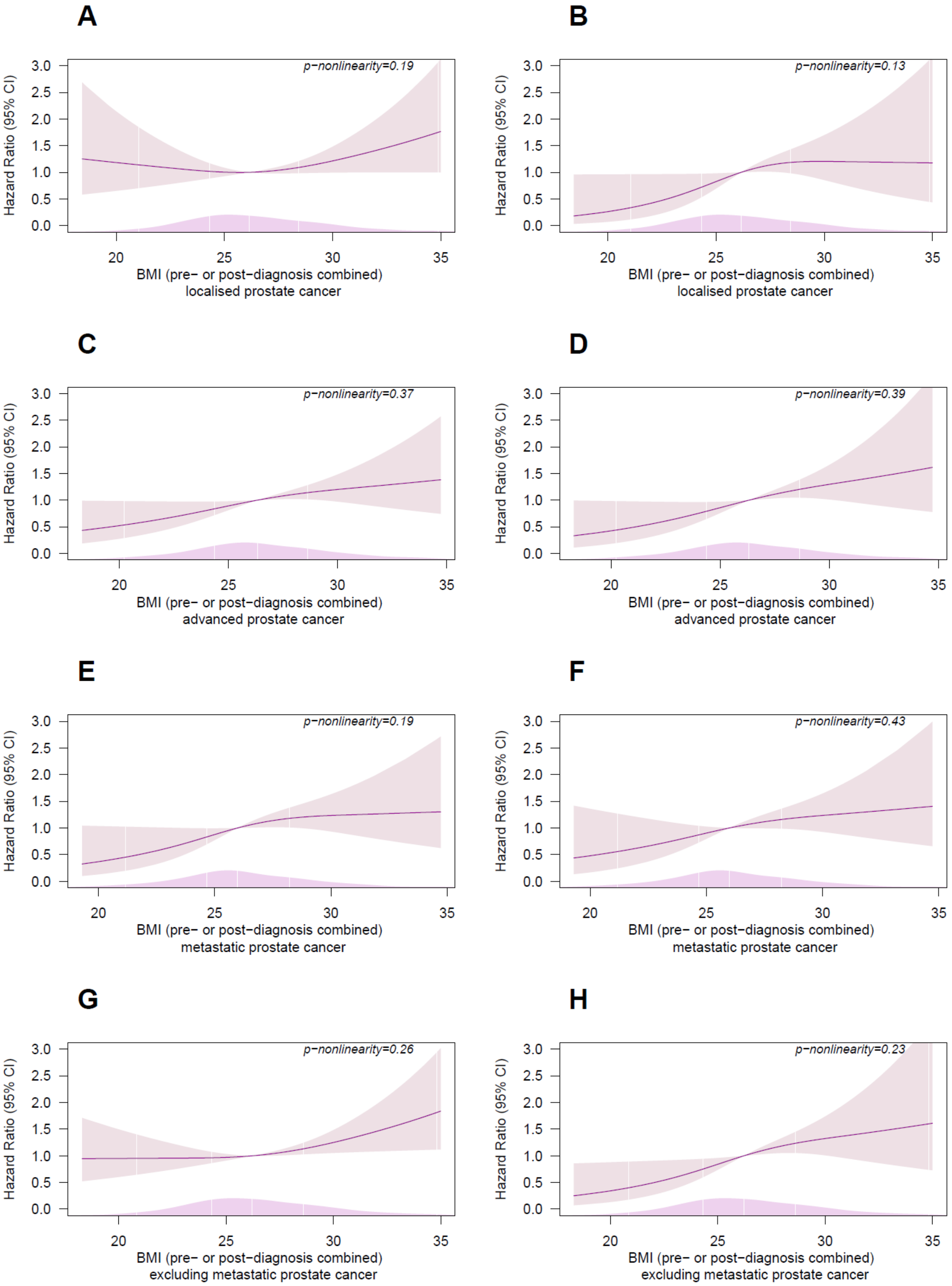

Supplementary Figure 3 – Subgroup analysis by prostate cancer grade.

Hazard ratios (HRs) from restricted cubic spline analysis, describing the association between BMI (kg/m^2) collected pre- or post-diagnosis combined in men with Gleason score 2-6/well-differentiated tumours and A) all-cause mortality (men/deaths=427/106) B) prostate cancer-specific mortality (men/deaths=427/34); in men with Gleason score 7/moderately differentiated tumours and C) all-cause mortality (men/deaths=359/118) D) prostate cancer-specific mortality (men/deaths=359/59); and in men with Gleason score 8-10/poorly-undifferentiated tumours and E) all-cause mortality (men/deaths=156/96) F) prostate cancer-specific mortality (men/deaths=156/70). HRs are based on the main Cox proportional hazards regression model of the present study adjusted for age (continuous), year of diagnosis (continuous), tumour stage (categorical) and smoking status (categorical) and stratified by EPIC country; knots at the 10th, 50th and 90th percentiles of BMI. Median BMI of the individuals included in analyses was used as referent: 26.4 kg/m^2 for those with Gleason 2-6, 25.9 kg/m^2 for those with Gleason 7 and 26.3 kg/m^2 in the analysis for Gleason 8-10. The smooth density plot represents the density of the population across the spline variable.

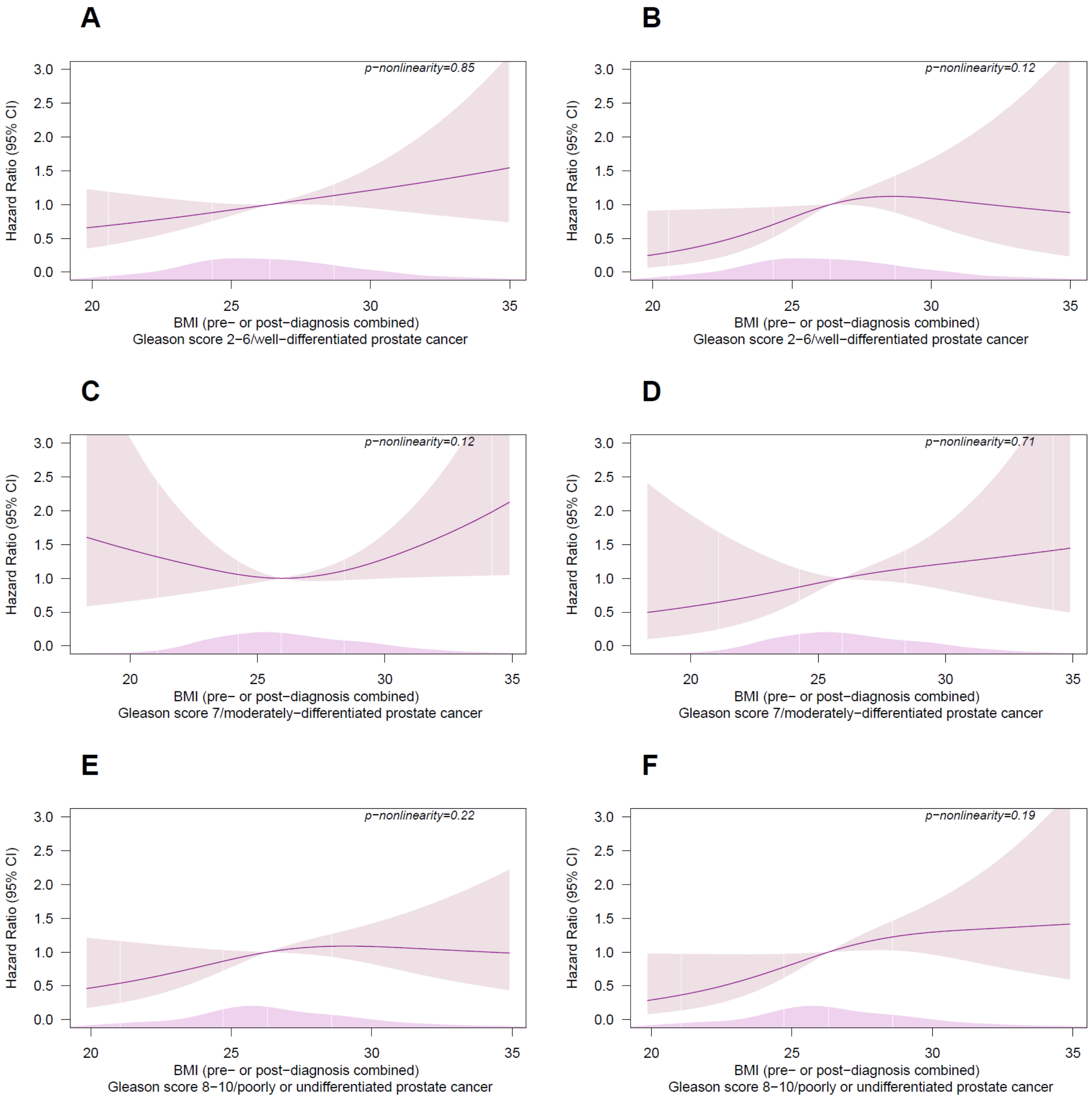

Supplementary Figure 4 – Lag-analysis of BMI (excluding deaths in the first year of follow-up).

Hazard ratios (HRs) from restricted cubic spline analysis describing the association between post-diagnosis BMI after excluding deaths within the first year of follow-up after the data collection/questionnaire and (A) all-cause mortality (men/deaths=363/117) and (B) prostate cancer-specific mortality(men/deaths=363/57); and for pre- or post-diagnosis BMI combined, after excluding deaths within the first year of follow-up after the data collection/questionnaire and (C) all-cause mortality (men/deaths=930/308) and (D) prostate cancer-specific mortality (men/deaths=930/155). HRs are based on the main Cox proportional hazards regression model of the present study adjusted for age (continuous), year of diagnosis (continuous), tumour stage (categorical), tumour grade (categorical) and smoking status (categorical) and stratified by EPIC country; knots at the 10th, 50th and 90th percentiles of BMI. Median BMI of the individuals included in analyses was used as referent: 25.9 kg/m^2 for those with post-diagnosis BMI, 26.2 kg/m^2 for those with pre- or post-diagnosis BMI. The smooth density plot represents the density of the population across the spline variable.

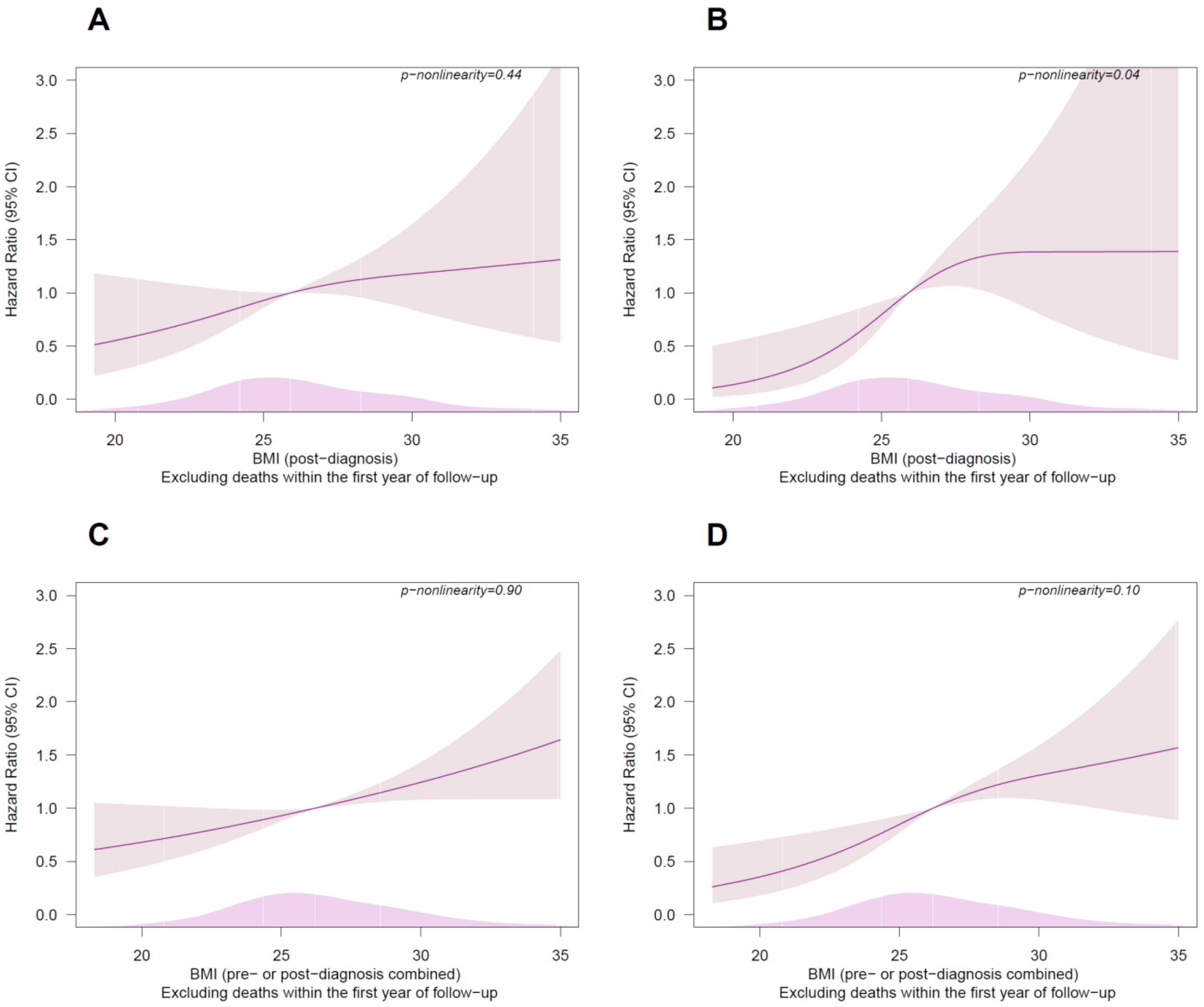

Supplementary Figure 5 – Analysis of post-diagnosis BMI adjusted for pre-diagnosis BMI.

Hazard ratios (HRs) from restricted cubic spline analysis, describing the association between post-diagnosis BMI adjusted for pre-diagnosis/baseline BMI, in addition to the other covariates with (A) all-cause mortality (men/deaths=372/126) and (B) prostate cancer-specific mortality (men/deaths=372/63). HRs are based on the main Cox proportional hazards regression model of the present study adjusted for age (continuous), year of diagnosis (continuous), tumour stage (categorical), tumour grade (categorical), smoking status (categorical), BMI (continuous) at baseline and stratified by EPIC country; knots at the 10th, 50th and 90th percentiles of BMI. Median BMI of the individuals included in analyses was used as referent: 25.9 kg/m^2. The smooth density plot represents the density of the population across the spline variable.

A

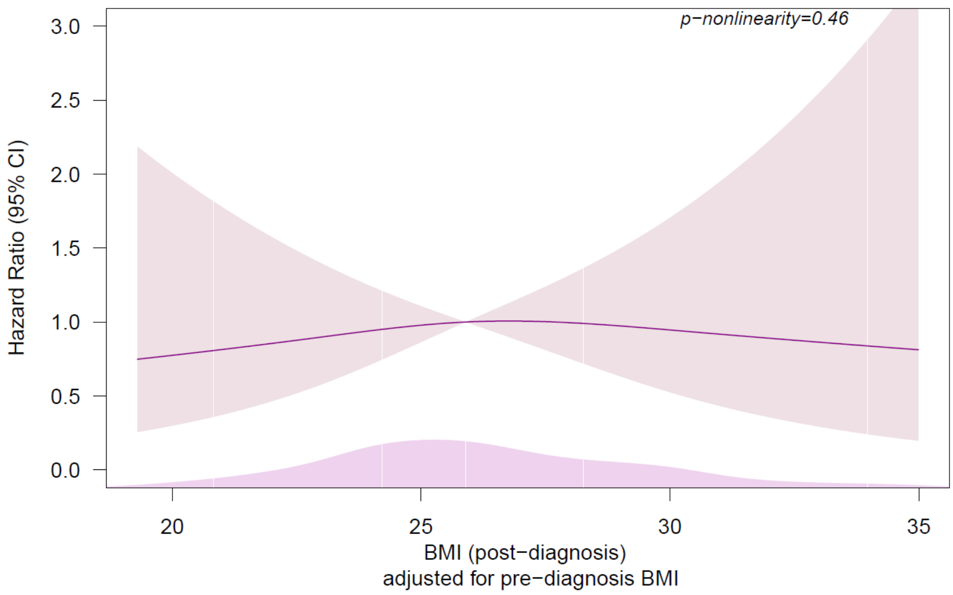

B

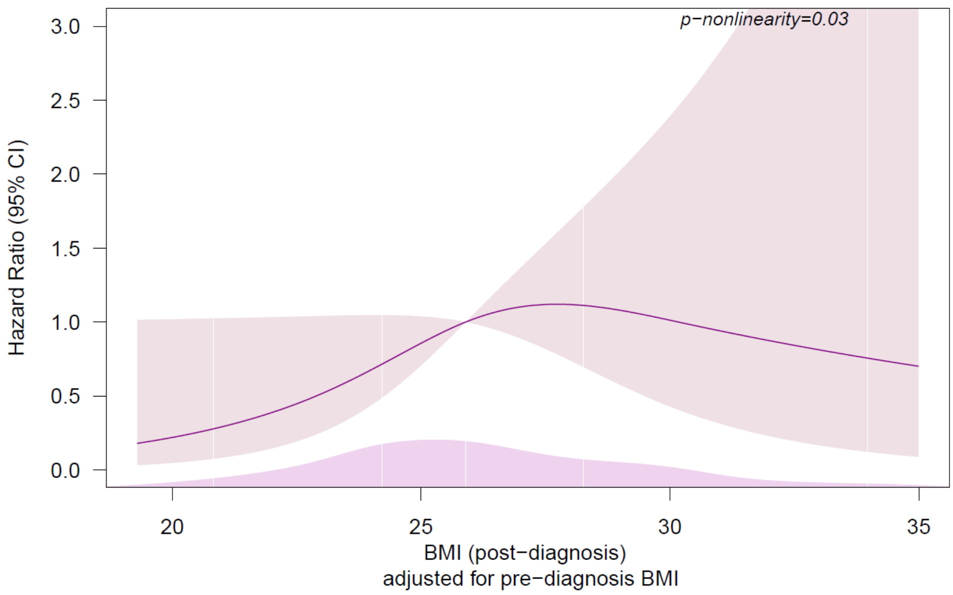

Supplementary Figure 6 – Total number of men with BMI data at one and two years before diagnosis, and at each year post-diagnosis up to five years after.
A. before and B. after exclusions for missing data in covariates.

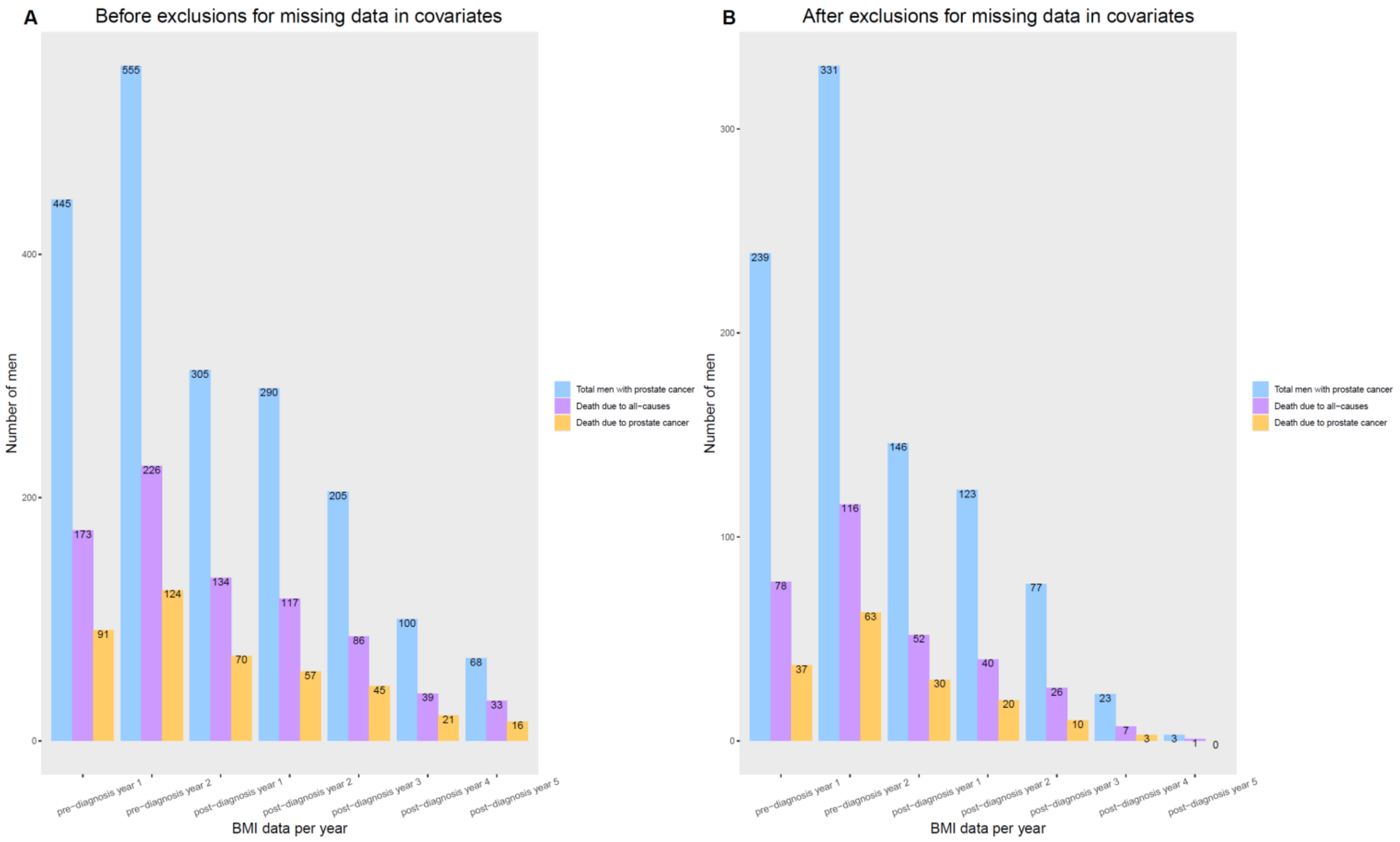

Supplementary Figure 7 – Analysis of BMI for each year pre-diagnosis (up to two years pre-diagnosis).

Hazard ratios (HRs) from restricted cubic spline analysis describing the association between BMI during the first-year pre-diagnosis and (A) all-cause mortality (men/deaths=239/78) and (B) prostate cancer-specific mortality (men/deaths=239/37); BMI during the second-year pre-diagnosis and (A) all-cause mortality (men/deaths=331/116) and (B) prostate cancer-specific mortality (men/deaths=331/63). HRs are based on the main Cox proportional hazards regression model of the present study adjusted for age (continuous), year of diagnosis (continuous), tumour stage (categorical), tumour grade (categorical) and smoking status (categorical) and stratified by EPIC country; knots at the 10th, 50th and 90th percentiles of BMI. Median BMI of the individuals included in analyses was used as referent: 26.2 kg/m^2 for those in the first-year pre-diagnosis BMI, 26.5 kg/m^2 for those in the second-year pre-diagnosis. The smooth density plot represents the density of the population across the spline variable.

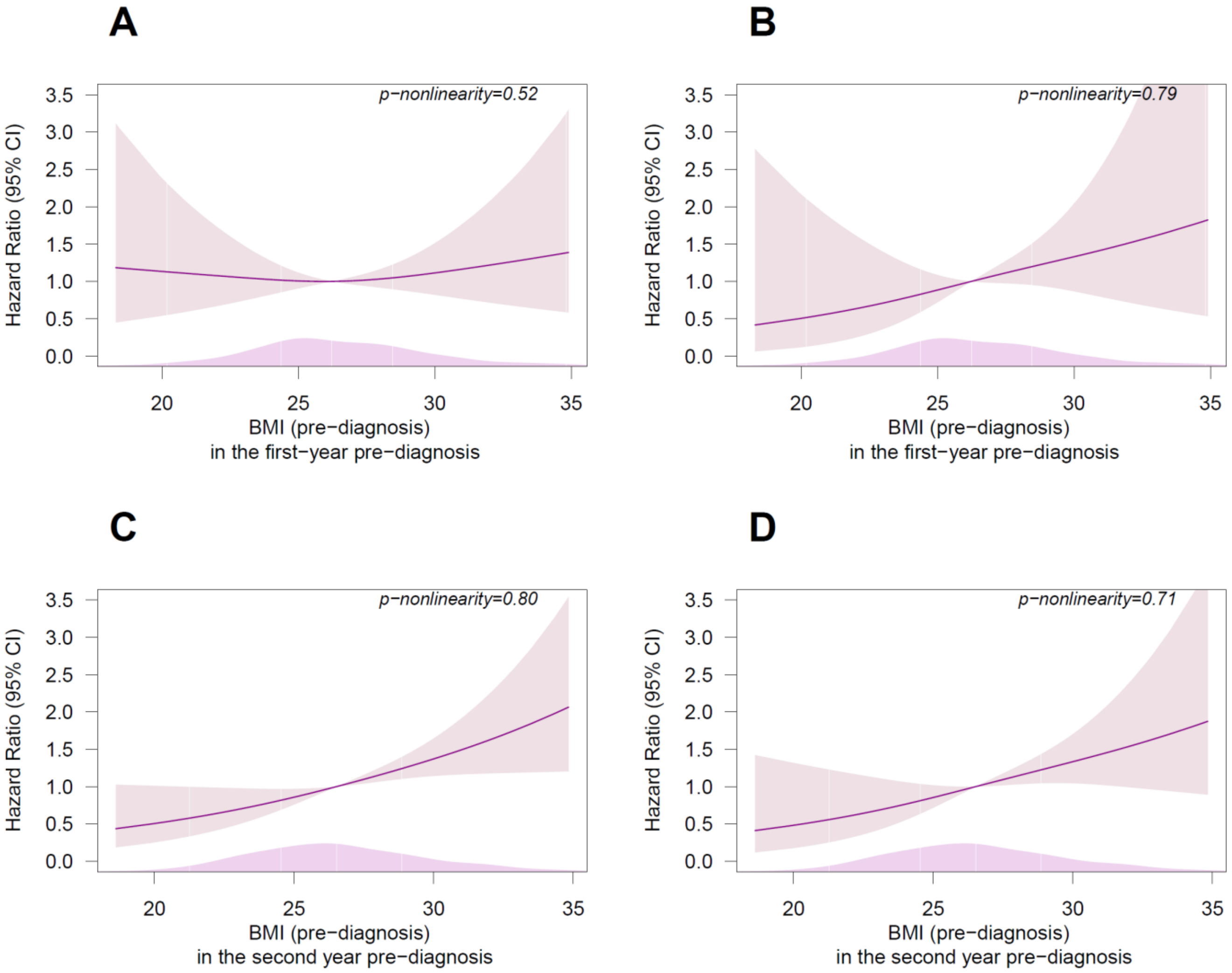

Supplementary Figure 8 – Analysis of BMI for each year post-diagnosis (up to the third-year post-diagnosis).

Hazard ratios (HRs) from restricted cubic spline analysis describing the association between post-diagnosis BMI in the first-year post-diagnosis and (A) all-cause mortality (men/deaths=146/52) and (B) prostate cancer-specific mortality (men/deaths=146/30); in the second-year post-diagnosis and (C) all-cause mortality (men/deaths=123/40) and (D) prostate cancer-specific mortality (men/deaths=123/20); in the third-year post-diagnosis and (E) all-cause mortality (men/deaths=77/26) and (F) prostate cancer-specific mortality (men/deaths=77/10). HRs are based on the main Cox proportional hazards regression model of the present study adjusted for age (continuous), year of diagnosis (continuous), tumour stage (categorical), tumour grade (categorical) and smoking status (categorical) and stratified by EPIC country; knots at the 10th, 50th and 90th percentiles of BMI. Median BMI of the individuals included in analyses was used as referent: 25.7 kg/m^2 for those in the first-year post-diagnosis BMI, 26.3 kg/m^2 for those in the second-year post-diagnosis and 26.2 kg/m^2 for the third-year post-diagnosis. Data was scarce beyond the third-year post-diagnosis (plots not shown). The smooth density plot represents the density of the population across the spline variable.

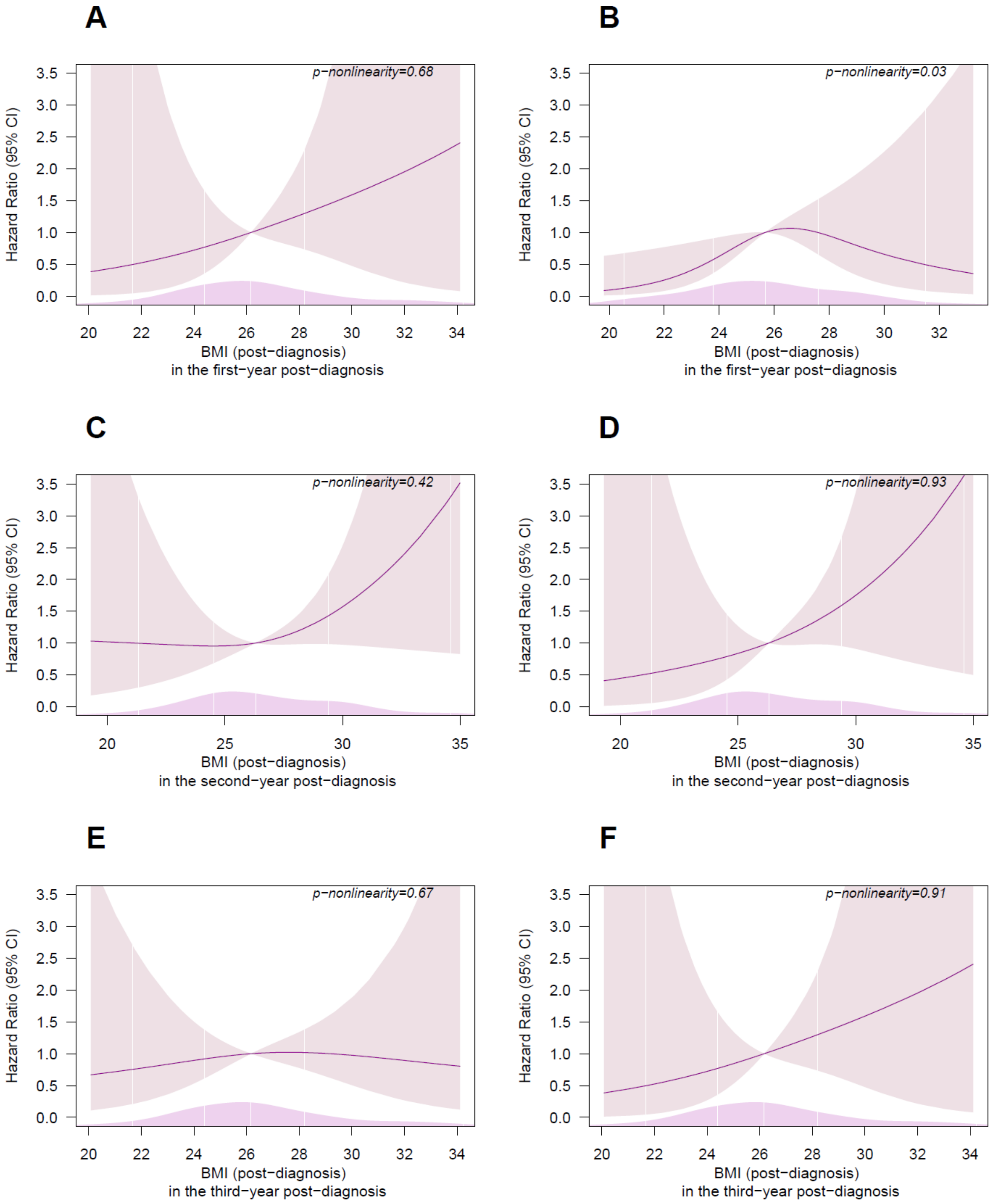

Supplementary Figure 9 – Stratified analysis by smoking status for pre- or post-diagnosis BMI combined.

Hazard ratios (HRs) from restricted cubic spline analysis describing the association between pre- or post-diagnosis BMI combined stratified by smoking status i.e., never smokers and (A) all-cause mortality (men/deaths=334/97) and (B) prostate cancer-specific mortality (men/deaths=334/56); current smokers and (C) all-cause mortality (men/deaths=135/74) and (D) prostate cancer-specific mortality (men/deaths=135/39); former smokers and (E) all-cause mortality (men/deaths=473/149) and (F) prostate cancer-specific mortality (men/deaths=473/68). HRs are based on the main Cox proportional hazards regression model of the present study adjusted for age (continuous), year of diagnosis (continuous), tumour stage (categorical) and tumour grade (categorical) and stratified by EPIC country; knots at the 10th, 50th and 90th percentiles of BMI. Median BMI of the individuals included in analyses was used as referent: 25.8 kg/m^2 for never smokers; BMI, 25.5 kg/m^2 for current smokers and 26.7 kg/m^2 for the former smokers. The smooth density plot represents the density of the population across the spline variable.

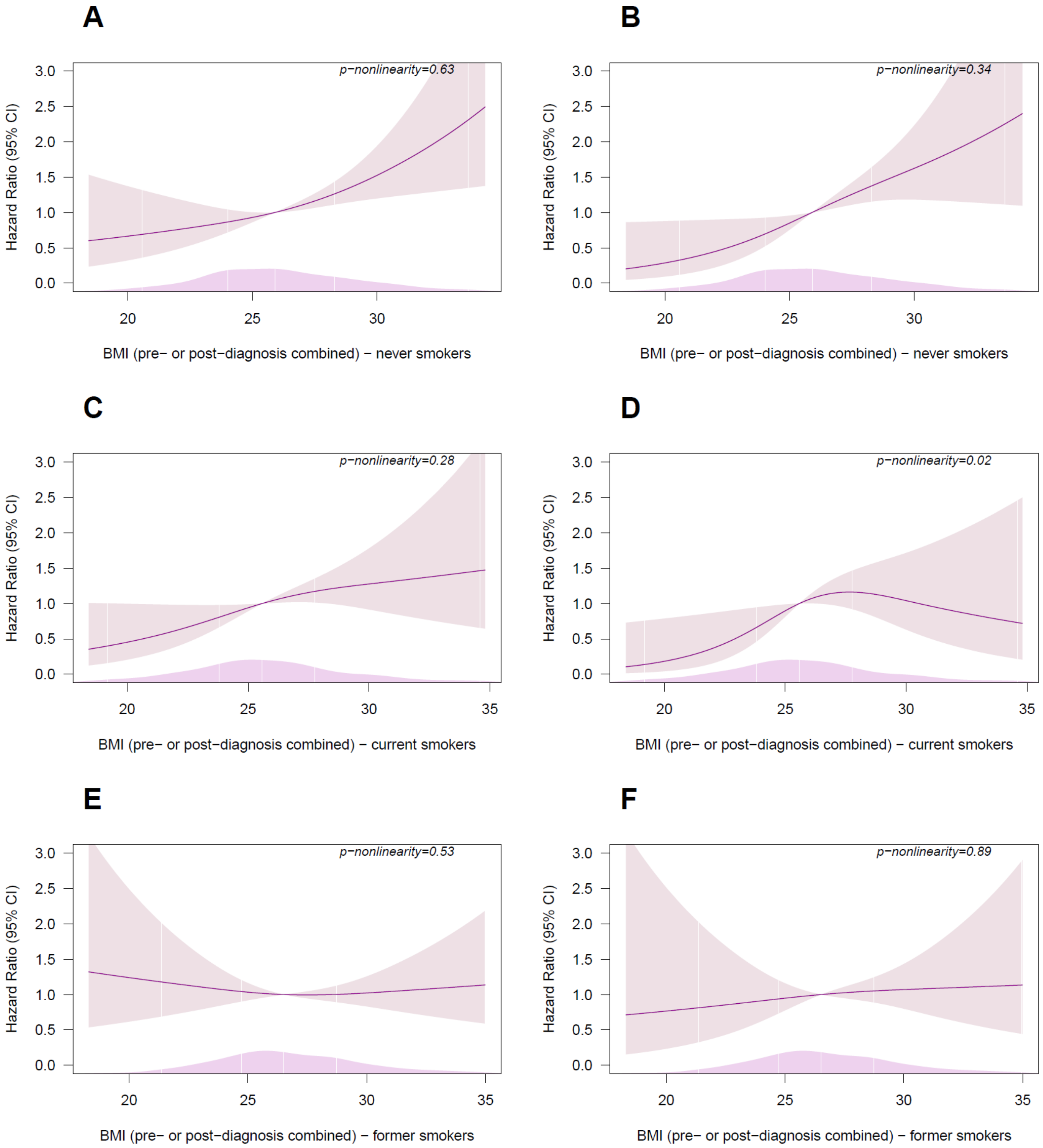

Supplementary Figure 10 – Additional adjustment for highest school level as proxy for socioeconomic status in the analysis of pre- or post-diagnosis BMI combined.

Hazard ratios (HRs) from restricted cubic spline analysis, describing the association between pre- or post-diagnosis BMI combined with additional adjustment for highest education level attained and (A) all-cause mortality (men/deaths=936/318) and (B) prostate cancer-specific mortality (men/deaths=936/163). HRs are based on the main Cox proportional hazards regression model of the present study adjusted for age (continuous), year of diagnosis (continuous), tumour stage (categorical), tumour grade (categorical), smoking status (categorical) and stratified by EPIC country; knots at the 10th, 50th and 90th percentiles of BMI. Median BMI of the individuals included in analyses was used as referent: 26.2 kg/m^2. The smooth density plot represents the density of the population across the spline variable.

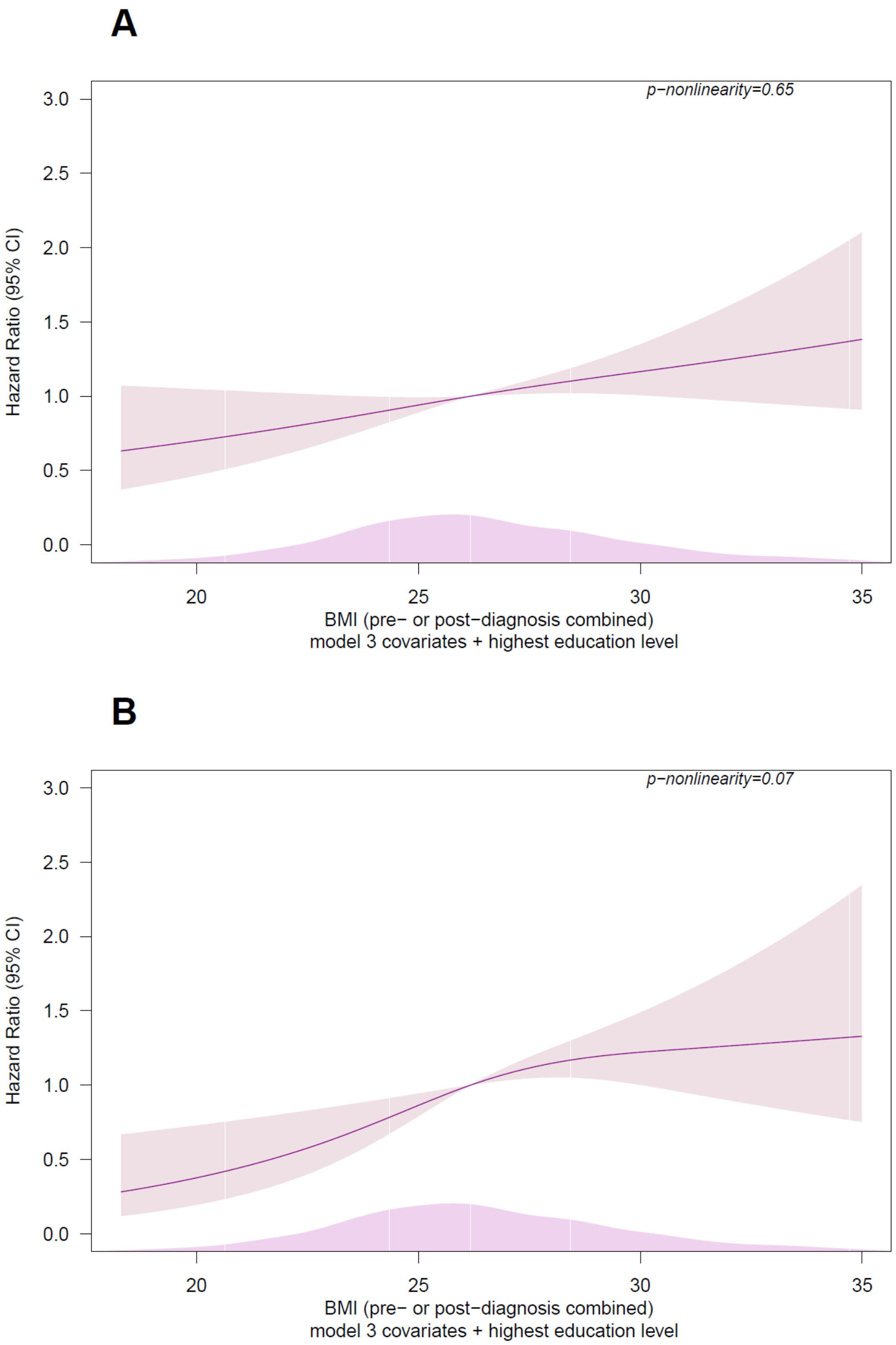

Supplementary Figure 11 – **Analysis of waist circumference and all-cause and prostate cancer-specific mortality.**
Hazard ratios (HRs) from Cox proportional hazards model with restricted cubic spline curves describing the association between waist circumference (cm) collected pre- or post-diagnosis combined and A) all-cause mortality (men/deaths=362/120) B) prostate cancer-specific mortality (men/deaths=362/79), pre-diagnosis waist circumference and C) all-cause mortality (men/deaths=245/86) D) prostate cancer-specific mortality (men/deaths=245/53), post-diagnosis waist circumference and E) all-cause mortality (men/deaths=117/34) F) prostate cancer-specific mortality (men/deaths=117/26). HRs are based on the main Cox proportional hazards regression model of the present study adjusted for age (continuous), year of diagnosis (continuous), tumour stage (categorical), tumour grade (categorical), smoking status (categorical) and smoking status (categorical) and stratified by EPIC country; knots at the 10th, 50th and 90th percentiles of waist circumference. Median waist circumference of the individuals included in analyses was used as referent: 97 cm in the pre- or post-diagnosis waist circumference analysis, 97 cm in the pre-diagnosis waist-circumference analysis and 98 cm in the post-diagnosis waist-circumference analysis. The smooth density plot represents the density of the population across the spline variable.

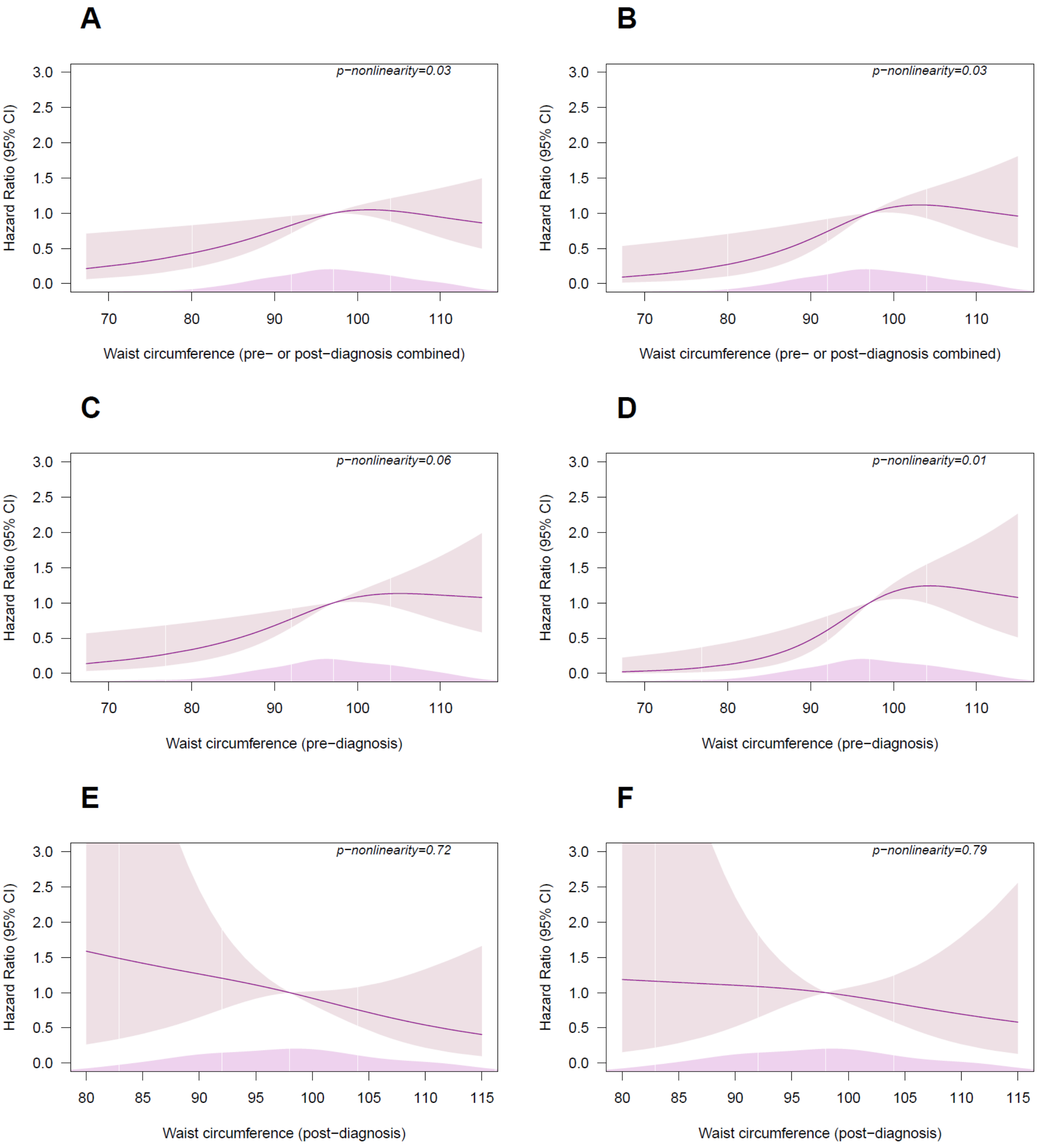

Supplementary Figure 12 – **Analysis of hip circumference and all-cause and prostate cancer-specific mortality.**

Hazard ratios (HRs) from Cox proportional hazards model with restricted cubic spline curves describing the association between hip circumference (cm) collected pre- or post-diagnosis combined and A) all-cause mortality (men/deaths=167/45) B) prostate cancer-specific mortality (men/deaths=167/23), pre-diagnosis hip circumference and C) all-cause mortality (men/deaths=128/42) D) prostate cancer-specific mortality (men/deaths=128/21). Plots not generated for post-diagnosis hip circumference and mortality outcomes due to limited data. HRs are based on the main Cox proportional hazards regression model of the present study adjusted for age (continuous), year of diagnosis (continuous), tumour stage (categorical), tumour grade (categorical), smoking status (categorical) and smoking status (categorical) and stratified by EPIC country; knots at the 10th, 50th and 90th percentiles of hip circumference. Median hip circumference of the individuals included in analyses was used as referent: 101 cm in the pre- or post-diagnosis hip-circumference analysis and 100 cm in the pre-diagnosis hip-circumference analysis. The smooth density plot represents the density of the population across the spline variable.

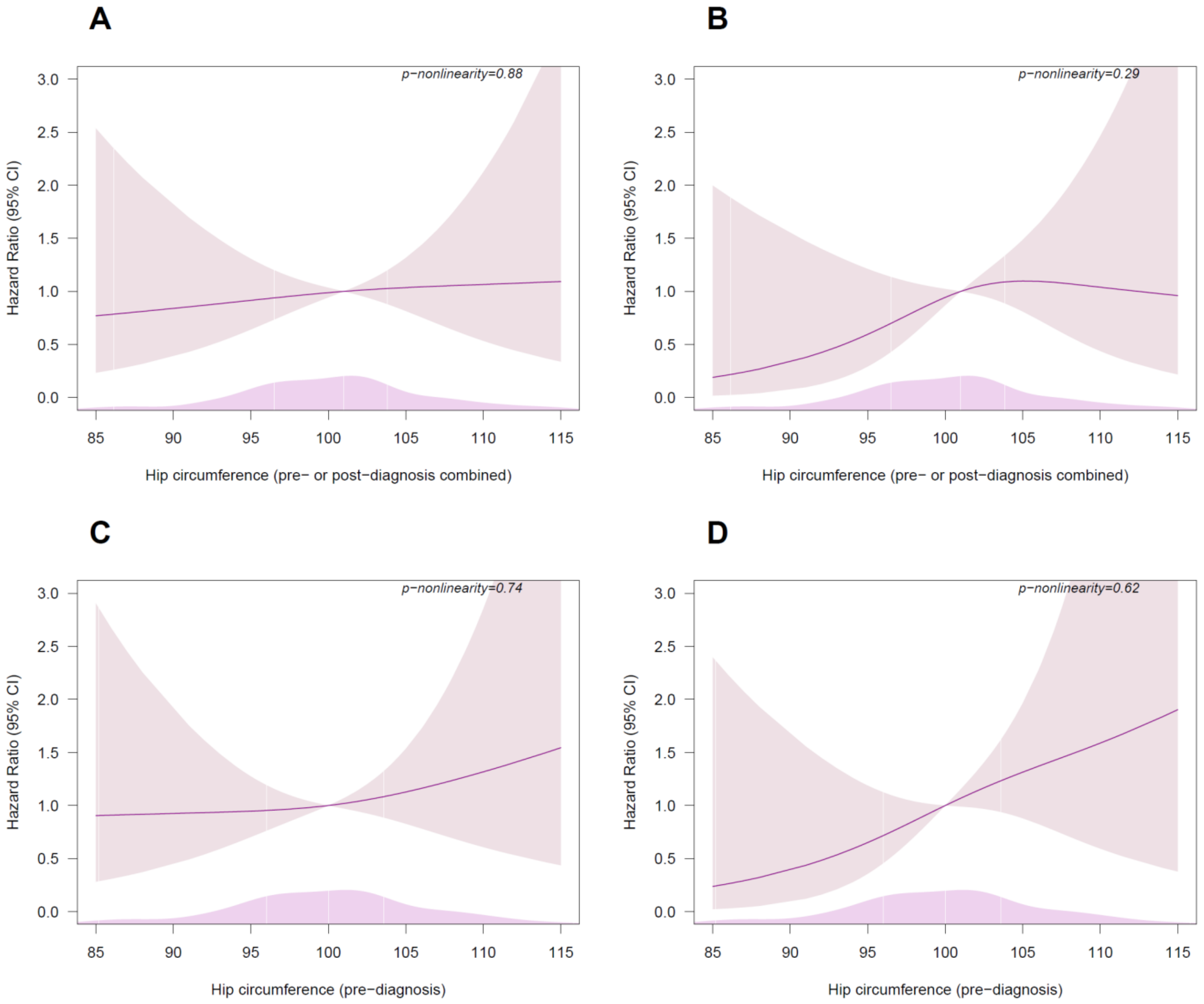

Supplementary Figure 13 – **Analysis of waist-to-hip ratio and all-cause and prostate cancer-specific mortality.**

Hazard ratios (HRs) from Cox proportional hazards model with restricted cubic spline curves, describing the association between waist-to-hip ratio collected pre- or post-diagnosis combined and A) all-cause mortality (men/deaths=167/45) B) prostate cancer-specific mortality (men/deaths=167/23), pre-diagnosis waist-to-hip ratio and C) all-cause mortality (men/deaths=128/42) D) prostate cancer-specific mortality (men/deaths=128/21). Plots not generated for post-diagnosis waist-to-hip ratio and mortality outcomes due to limited data. HRs are based on the main Cox proportional hazards regression model of the present study adjusted for age (continuous), year of diagnosis (continuous), tumour stage (categorical), tumour grade (categorical), smoking status (categorical) and smoking status (categorical) and stratified by EPIC country; knots at the 10th, 50th and 90th percentiles of waist-to-hip ratio. Median waist-to-hip of the individuals included in analyses was used as referent: 0.96 units in the pre- or post-diagnosis analysis and 0.97 units in the pre-diagnosis analysis. The smooth density plot represents the density of the population across the spline variable.

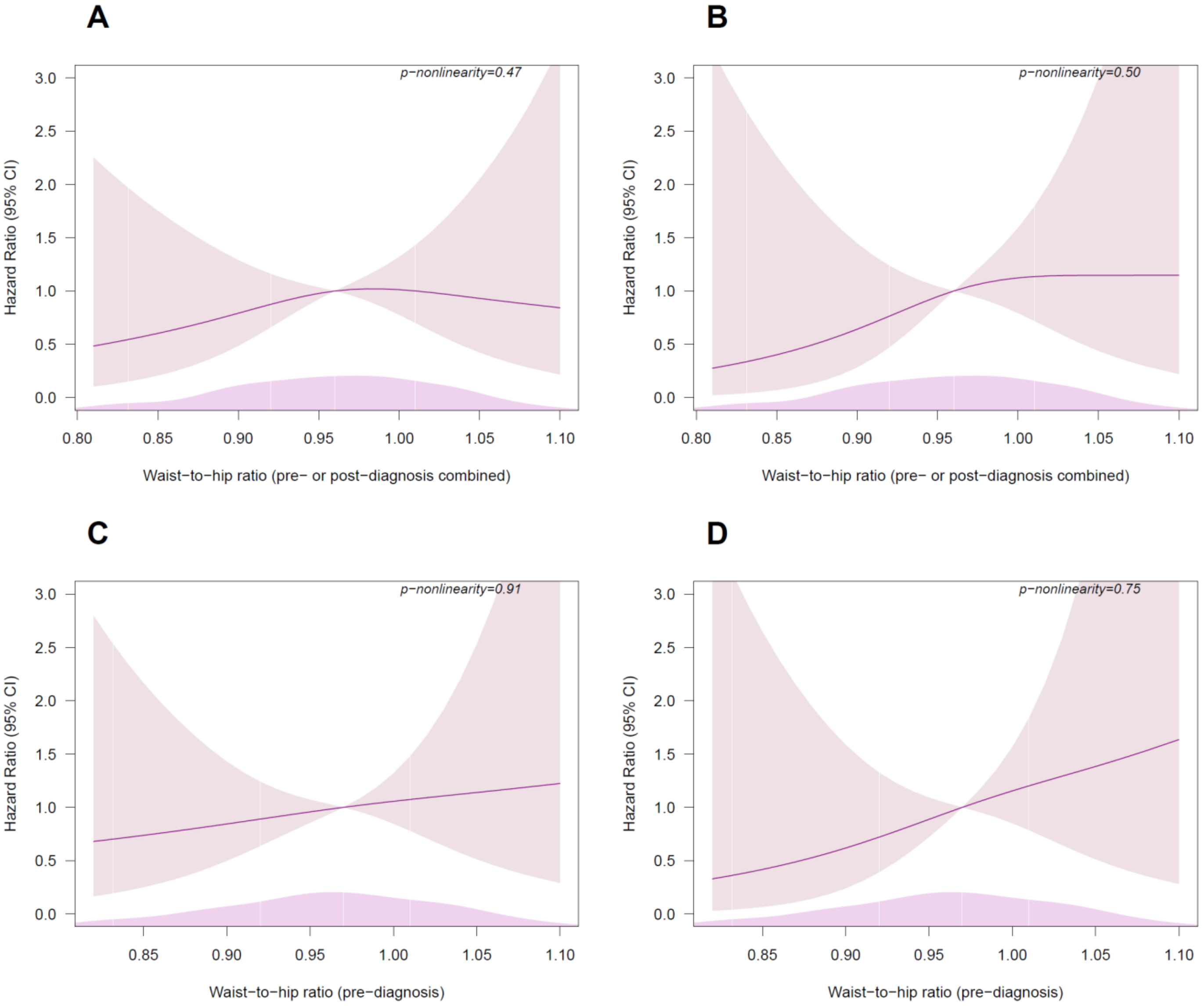

Supplement: pkae070_Supplementary_Data [file pkae070_supplementary_data.pdf]
